# Involvement of Myeloid Cells and Noncoding RNA in Abdominal Aortic Aneurysm Disease

**DOI:** 10.1089/ars.2020.8035

**Published:** 2020-08-13

**Authors:** Christoph Knappich, Joshua M. Spin, Hans-Henning Eckstein, Philip S. Tsao, Lars Maegdefessel

**Affiliations:** ^1^ Department for Vascular and Endovascular Surgery, Klinikum rechts der Isar, Technical University of Munich, Munich, Germany.; ^2^ Division of Cardiovascular Medicine, Stanford University School of Medicine, Stanford, California, USA.; ^3^ Department of Medicine, Karolinska Institute, Stockholm, Sweden.

**Keywords:** aortic aneurysm, myeloid cell, monocyte, macrophage, noncoding RNA, microRNA

## Abstract

***Significance:*** Abdominal aortic aneurysm (AAA) is a potentially fatal condition, featuring the possibility of high-mortality rupture. To date, prophylactic surgery by means of open surgical repair or endovascular aortic repair at specific thresholds is considered standard therapy. Both surgical options hold different risk profiles of short- and long-term morbidity and mortality. Targeting early stages of AAA development to decelerate disease progression is desirable.

***Recent Advances:*** Understanding the pathomechanisms that initiate formation, maintain growth, and promote rupture of AAA is crucial to developing new medical therapeutic options. Inflammatory cells, in particular macrophages, have been investigated for their contribution to AAA disease for decades, whereas evidence on lymphocytes, mast cells, and neutrophils is sparse. Recently, there has been increasing interest in noncoding RNAs (ncRNAs) and their involvement in disease development, including AAA.

***Critical Issues:*** The current evidence on myeloid cells and ncRNAs in AAA largely originates from small animal models, making clinical extrapolation difficult. Although it is feasible to collect surgical human AAA samples, these tissues reflect end-stage disease, preventing examination of critical mechanisms behind early AAA formation.

***Future Directions:*** Gaining more insight into how myeloid cells and ncRNAs contribute to AAA disease, particularly in early stages, might suggest nonsurgical AAA treatment options. The utilization of large animal models might be helpful in this context to help bridge translational results to humans.

## Introduction

The prevalence of unhealthy diets, smoking, and inactive lifestyles in conjunction with an aging society has laid the cornerstones for cardiovascular diseases being a major public health burden in the developed world. Whereas atherosclerotic changes in arterial walls cause plaque formation and narrowing of the lumen, associated mechanisms may promote dilation at susceptible sites of the arterial tree. Arterial enlargement over 150% of the norm is defined an aneurysm, with abdominal aortic aneurysm (AAA) being typically defined as an infrarenal portion of the aorta wider than 3.0 cm.

**FIG. 1.**
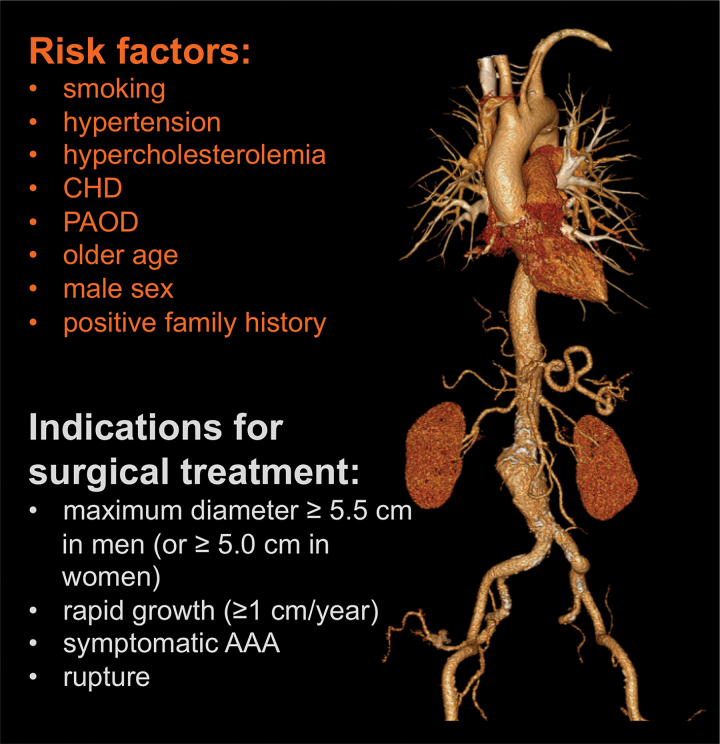
**AAA in spotlight.** Computed tomography angiogram of the thoracoabdominal arterial tree presenting with an infrarenal AAA. Presence of concomitant atherosclerotic calcifications predominantly in the regions of the aortic neck and the iliac arteries is frequent and attributable to similar risk factors. AAA, abdominal aortic aneurysm; CHD, coronary heart disease; PAOD, peripheral arterial occlusive disease. Color images are available online.

Although not considered to be merely a variant of atherosclerosis, AAA risk factors are similar, and include modifiable risks (smoking, hypertension, hypercholesterolemia, coronary heart disease, and peripheral arterial occlusive disease [PAOD]), and nonmodifiable risks (older age, male sex, and a positive family history) (Fig. 1) (55). Lower risks are seen in African American, Asian, and Hispanic patients as opposed to Caucasians, and with an active lifestyle combined with a healthy diet containing fruits, vegetables, and nuts. Counterintuitively, patients with diabetes mellitus are also at lower risk (55).

Possibly due to a decline in smoking, the prevalence of AAA has been decreasing in recent years to ∼2.2% in a cohort of 65-year old men (64, 129). Nevertheless, the natural course of disease is progressive with a risk of potentially lethal rupture. Mortality of ruptured AAA was described as high as 97.2% if untreated, decreased to 37.3% and 24.7% when treated by open surgical repair (OSR) or endovascular aortic repair (EVAR), respectively (50).

To date, available therapy is aimed at rupture prevention. Although recommended to reduce cardiovascular events (4), antiplatelet medications and antihypertensive agents have not been shown to influence AAA growth or rupture risk. Although a large systematic review found a possible association of statins with a reduction in AAA growth and rupture (114), similar effects have not been shown for other medication classes including angiotensin converting enzyme inhibitors, beta blockers, and doxycycline (41, 58). Accordingly, screening for AAA in populations at risk (23), surveillance of small AAAs, and pre-emptive surgical repair at the point when risk of rupture outweighs the surgical risks are crucial.

The maximum diameter of the aorta has been the most commonly employed reliable predictor for rupture. Whereas AAAs sized smaller than 5.5 cm in male patients were found to possess an annual rupture rate ∼1% (102), the rupture risk increases significantly beyond this threshold (66).

Based on these data, current guidelines recommend surgical treatment of AAAs if the maximum diameter is at least 5.5 cm in men (or 5.0 cm in women). Surgery is also recommended for rapidly progressing AAAs (≥1 cm/year), symptomatic AAAs, ruptured AAAs, and those with an excentric or saccular configuration (16, 142).

**FIG. 2.**
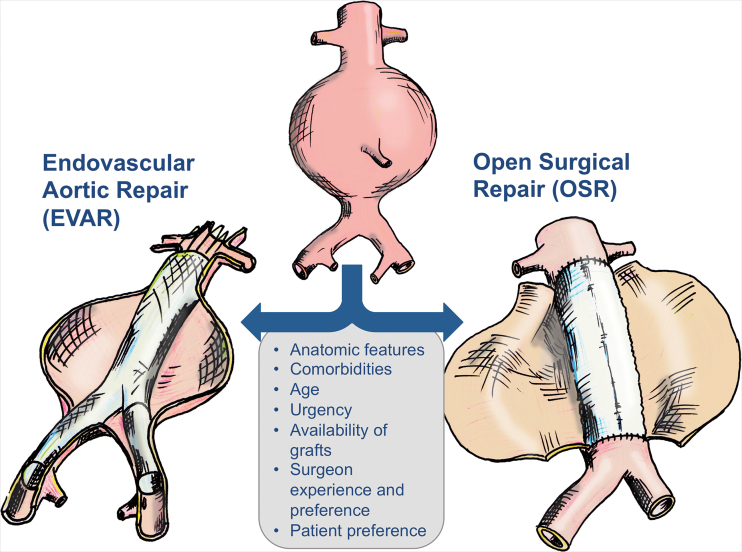
**Decision-making and therapeutic options for AAA**. Color images are available online.

Two surgical techniques coexist today (Fig. 2). OSR involves clamping of the infrarenal aorta and replacement by an alloplastic aorto-aortal tube graft or aorto-bi-iliac bifurcated graft. Since the early 1990s, EVAR has represented a minimally invasive option using a stent graft, which is inserted through the groin arteries. In EVAR, sealing is uniquely accomplished by the radial force and additional mechanisms (*i.e*., hooks and barbs) of the device. Insufficient sealing at the proximal or distal sealing zone, or inverse blood flow in branches emerging from the aorta, will lead to endoleaks, defined as persistent perfusion of the aneurysm sac. Delayed endoleaks may also occur, requiring life-long surveillance. Depending on the type of endoleak, reinterventions may become necessary.

Whereas initial results from randomized controlled trials found lower perioperative mortality rates after EVAR (0.5%–1.7%) compared with OSR (3%–4.7%) (43, 65, 103), long-term follow-up revealed a loss of the survival benefit over the first 2 years (98, 138). Furthermore, the reintervention rate and aneurysm-related mortality were found to be higher after EVAR than after OSR (98).

In light of the perioperative mortality and morbidity inherent to OSR and the long-term risks linked to EVAR (including the associated exposure to radiation during the procedure and follow-up), alternative options to treat or to prevent the occurrence of AAAs are desirable. A prerequisite for the evolution of treatment options targeting early stages of the disease, thereby preventing AAA development, is to understand the underlying pathomechanisms. This review aims to give an overview on the role of myeloid cells and noncoding RNAs (ncRNAs) as potential targets for future therapeutic approaches to cure AAA disease. With respect to ncRNAs, emphasis is placed on those species involved in inflammatory processes.

## The Role of Myeloid Cells in AAA

Various immune cells including monocytes, macrophages, T and B cells, mast cells, neutrophils, and dendritic cells have been demonstrated to be involved in AAA formation (26).

### Circulating blood monocytes

Hematopoiesis in the bone marrow involves differentiation of monocytes from multipotent hematopoietic stem cells. Monocytes are the largest leukocytes circulating in the blood stream and can differentiate into macrophages.

Various subsets of monocytes with different morphology, surface markers, gene expression profiles, and functions have been described. Based on the expression of CD14 and CD16 as cell surface markers, they can be classified into three main subsets (Table 1). The major population of monocytes (∼90%) is defined as classical monocytes, which are characterized by high surface level expression of CD14 (CD14^++^CD16^−^). The minor population of cells with low expression of CD14 and high expression of CD16 (CD14^+^CD16^++^) is defined as nonclassical monocytes. Monocytes in between these two subsets are defined as intermediate monocytes (CD14^++^CD16^+^) (160).

**Table 1.** Myeloid Cell Subsets and Their Involvement in Abdominal Aortic Aneurysm Disease

Cell subset	Surface markers	Stimulating factors	Pathway	Secreted products	Function	References
Monocytes						
Classical	CD14^++^CD16^−^	CCL2/CCR2		IL-1	Proinflammatory	(160)
Nonclassical	CD14^+^CD16^++^	NR4A1, CX_3_CL1/CX_3_CR1		IL-10, TGF-β	Tissue repair, patrolling	(131)
Intermediate	CD14^++^CD16^+^	CCL2/CCR2, CCL5/CCR5		IL-1, TNF-α	Proinflammatory	(146, 160)
Macrophages						
M1	CD80, CD86, CD16, CD14, CD68	TNF-α, IFN-γ, LPS, NF-κB	STAT1, AP-1, NF-κB	TNF-α, IL-6, IL-1β, iNOS, MCP-1	Proinflammatory, cytotoxicity	(131)
M2	CD206, CD163, CD68	IL-4, IL-13, IL-10, TGF-β	STAT6, PPAR-γ, CREB	Arg1, Ym1	Anti-inflammatory, tissue remodeling, tissue repair	(131)
Lymphocytes						
Th1	CD4	IL-12, IFN-γ	STAT4, T-bet	IFN-γ, TNF-α, IL-2	Activation of macrophages, recruitment of proinflammatory cells	(159)
Th2	CD4	IL-2, IL-4	STAT6, GATA-3	IL-4, IL-5, IL-10, IL-13, IL-25	Limitation of macrophage cytotoxicity, regulation of MMPs	(26, 116, 159)
Th17	CD4	IL-1, IL-6, IL-21, IL-23, TGF-β	STAT3, RORγt	IL-17A, IL-17F, IL-21, IL-22	Promotion of macrophage recruitment	(26, 159)
Th_reg_	CD4	IL-2, TGF-β	STAT5, Foxp3	IL-10, IL-35, TGF-β	Limitation of T_eff_ proliferation, reducing TNF-α and IFN-γ secretion from T_eff_; removing of autoreactive T cells	(26, 159)
B	CD19, CD20, CD21, CD22, CD24, CD72	Non-nucleic acid components of microbes, bacterial LPS/flagellin, dsRNA, ssRNA	MyD88, IRAK	IL-2, IL-4, IL-6, IL-10, IFN-γ, TNF-α	Activation of complement cascade, promoting MMP expression, recruitment of proinflammatory cells	(63, 84, 134)

CCR2, C-C motif chemokine receptor 2; CX_3_CR1, C-X_3_-C motif chemokine receptor 1; dsRNA, double-stranded RNA; Foxp3, forkhead box P3; IFN-γ, interferon gamma; LPS, lipopolysaccharide; MMPs, matrix metalloproteinases; NF-κB, nuclear factor kappa-light-chain-enhancer of activated B cells; RORγt, receptor-related orphan receptor γt; ssRNA, single-stranded RNA; STAT, signal transducer and activator of transcription; T_eff_, T effector; TGF-β, transforming growth factor beta; TNF-α, tumor necrosis factor alpha.

Different expression of surface antigens determines the various functions of the three monocyte populations. Classical monocytes express a broad range of sensing receptors and proteins involved in tissue repair and immune responses. Furthermore, high-level expression of proinflammatory genes (*e.g*., S100A12 and S100A8/9) suggests their ability to support inflammation (146). The nonclassical subset of monocytes expresses high levels of proteins involved in cytoskeleton rearrangement, which might contribute to their patrolling behavior (24) and FcR-mediated phagocytosis (146). Beyond this, nonclassical monocytes produce high levels of tumor necrosis factor alpha (TNF-α) and IL-1β in response to lipopolysaccharide (LPS) (146) or after activation with Toll-like receptor ligands (7).

Intermediate monocytes seem to possess superior T cell stimulatory functions, which might originate from their high MHC class II expression (146).

The involvement of monocytes in AAA disease has been suggested by a number of studies. AAA patients have higher proportions of intermediate blood monocytes than healthy individuals. However, the proportion of circulating classical monocytes is lower in these patients (40).

Monocyte-derived macrophages in AAA patients differ from those in PAOD patients in terms of protein and gene expression, suggesting specific involvement in AAA disease. Those derived from AAA patients showed differences in expression of proteins related to extracellular matrix (ECM; *e.g*., beta-actin and fibronectin) and inflammation (*e.g*., tissue inhibitor of metalloproteinases [TIMP]-3) (61).

Alterations to monocytes have also been shown in murine AAA models. After infusion of angiotensin II (AngII) for 2 weeks, ApoE^−/−^ (apolipoprotein E) mice reacted with an increase in circulating classical CCR2^+^ monocytes, which seem to play a role in the inflammatory reaction contributing to AAA formation (90). Similar results showed elevated monocyte levels [lymphocyte antigen (LY)6C^high^ and lymphocyte antigen LY6C^low^ monocytes] after AngII infusion in mice that later developed AAA. These monocyte subsets were shown to have been mobilized from the spleen rather than the bone marrow. Splenectomy led to a reduction of aortic macrophages. Mice with unchanged monocyte levels did not present with later AAA formation (86). When ApoE^−/−^ mice were studied as an atherosclerosis model, LY6C^high^ monocytes were shown to dominate the population in the blood stream (130). In contrast, within the arterial wall, LY6C^low^ (corresponding to nonclassical monocytes in humans) monocytes are more frequently seen to develop into plaque cells, and express the dendritic cell-associated marker CD11c (132).

Although LY6C^high^ monocytes require C-C motif chemokine receptor 2 (CCR2), CCR5, and C-X_3_-C motif chemokine receptor 1 (CX_3_CR1) to enter the arterial wall, recruitment of LY6C^low^ monocytes seems to partially rely on CCR5, but not on CX_3_CR1 (132). Notably, combined inhibition of CCL2, CX_3_CR1, and CCR5 using ApoE^−/−^/CCL2^−/−^/CX_3_CR1^−/−^ triple knockout mice together with Met-CCL5 (an antagonist of CCR5 signaling) resulted in significantly reduced atherosclerotic lesion size and near-complete abrogation of macrophage accumulation (19).

Furthermore, monocyte binding to the AngII-infused aorta was dependent on CD14 (8). Despite contradictory findings on subset predominance between human data and animal models, it is widely agreed that circulating blood monocytes may contribute to the pathogenesis of AAA. Although human studies found an increase of intermediate nonclassical monocytes to the expense of classical monocytes in AAA patients (40, 112), mouse models also revealed an increase of classical monocytes (86, 90). Leaving out the fact that animal models are prone to confounders, this difference might be due to different phases of AAA disease. Although mouse models regularly display the early stages of AAA development, studying humans with fully evolved AAAs depicts the end stage of a chronic disease. Conversion of monocyte subsets from the classical toward nonclassical subsets over the time course of disease was demonstrated for other conditions (*e.g*., myocardial infarction and rheumatoid arthritis) (59). A similar polarization toward a more mature monocyte phenotype during AAA development is possible.

### Macrophages

**FIG. 3.**
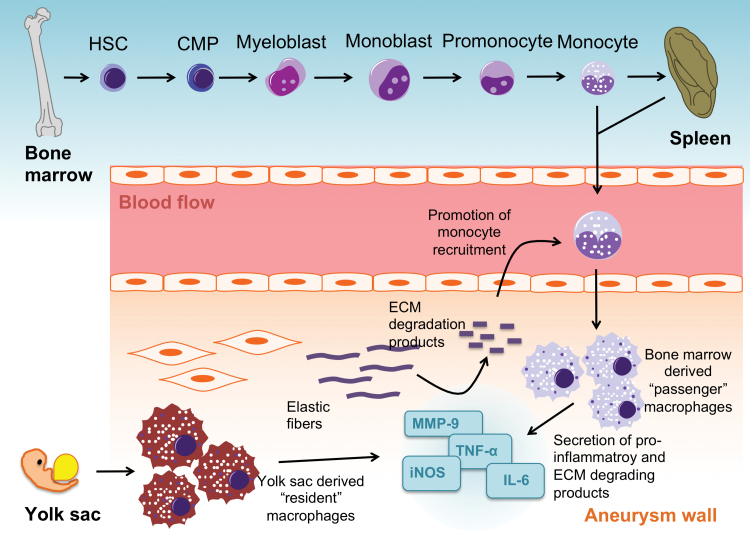
**Different origins and modes of action of tissue macrophages in AAA.** Myelopoiesis in the bone marrow is the source of circulating blood monocytes with the spleen acting as a reservoir. Extravasation of “passenger” monocytes into the aneurysm wall is a CD14-dependent mechanism. In parallel, a pool of “resident” tissue macrophages exists, which originate from the yolk sac and migrate into aortic tissue during embryonic development. During the early phase of AAA development, a shift toward M1 macrophages entails increased secretion of proinflammatory cytokines, including TNF-α, IL-6, IL-1β, iNOS, MCP-1, and ECM degrading products such as MMP-9. The inflammatory response within the aneurysm wall is a self-perpetuating system, as degradation products act as chemokines to attract further monocytes to extravasate. CMP, common myeloid progenitor; ECM, extracellular matrix; HSC, hematopoietic stem cell; MMP, matrix metalloproteinase; TNF-α, tumor necrosis factor alpha. Color images are available online.

Previous dogma stated that tissue macrophages originated exclusively from bone marrow-derived “passenger” monocytes, and extravasated from the blood stream into the aortic wall. More recent studies suggest that, in parallel, a pool of “resident” tissue macrophages exists (Fig. 3). These originate from the yolk sac, migrate into tissues during embryonic development, and can persist throughout adult life by local proliferation (100). Fate-mapping experiments have shown that arterial macrophages can arise embryonically from CX_3_CR1^+^ precursors and from bone marrow-derived monocytes that colonize the tissue immediately after birth (34). An additional source of macrophages might be phenotypic switching of smooth muscle cells (SMCs). In a mouse model it was demonstrated that some lineage-traced SMCs in advanced atherosclerotic lesions lacked expression of typical SMC markers like ACTA2 (140) and instead expressed macrophage markers (*e.g*., LGALS3) (118). Furthermore, cholesterol loading of mouse aortic SMCs resulted in a decrease of SMC-related genes (*e.g*., SM alpha-actin, alpha-tropomyosin, myosin heavy chain), whereas expression of macrophage-related genes (CD68, Mac-2, adenosine triphosphate-binding cassette transporter A1 [ABCA1]) was increased (111). However, despite their immunohistochemical resemblance, the functional properties of cholesterol-loaded SMCs and macrophages seem to differ. After incubation with 1 μm latex beads, cholesterol-loaded SMCs showed significantly less phagocytic activity compared with macrophages. Furthermore, SMC-derived macrophage-like cells were shown to possess less efferocytotic activity compared with macrophages (139).

Although a transdifferentiation of SMCs toward a macrophage-like phenotype was demonstrated in atherosclerotic disease (42) rather than in AAA, similar mechanisms are likely to be present.

**FIG. 4.**
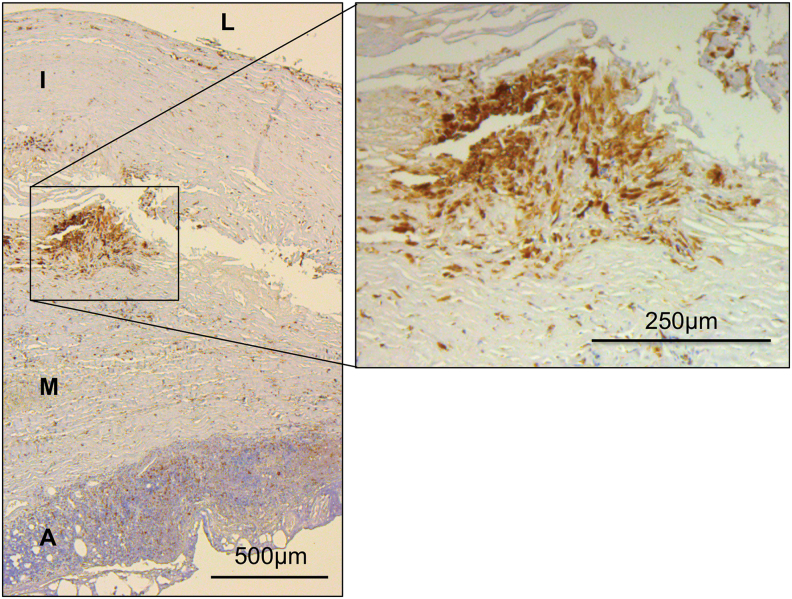
**Immunohistochemical staining of macrophages in human AAA tissue.** Macrophages and other CD68^+^ cells (*i.e*., SMC-derived macrophage-like cells) stained with anti-CD68 antibodies. Note CD68^+^ cells are accumulating mainly in the border region between tunica intima and tunica media, whereas lymphocytes are predominantly located in the adventitia forming the VALT. A, adventitia; I, tunica intima; L, vessel lumen; M, tunica media; SMC, smooth muscle cell; VALT, vascular-associated lymphoid tissue. Color images are available online.

In AAA specimens, accumulation of macrophages is found predominantly in the adventitia (30) and the intraluminal thrombus (108). Which population primarily fuels this increase of macrophages in AAA needs further investigation (106). Figure 4 shows immunohistochemical staining of human AAA tissue using anti-CD68 antibodies. As already outlined, macrophages and other CD68^+^ cells (*i.e*., SMC-derived macrophage-like cells) cannot be distinguished with this technique.

Macrophages are typically classified into M1 and M2 phenotypes (Table 1) (91, 131). *In vitro*, M1 macrophages are usually induced by two substances: interferon gamma (IFN-γ), which originates from natural killer cells *in vivo*, and LPS, which is a component of Gram-negative bacteria cell walls (131). Triggering macrophages with the mentioned stimuli causes production of proinflammatory cytokines and chemokines (*e.g*., IL-1β, IL-12, TNF-α, MCP-1, CCL2, and iNOS), all of which play a role in accelerating inflammation and killing pathogens (131). Alternatively activated macrophages (*e.g*., those formed after incubation with IL-4 and IL-13) are classified as M2. This phenotype produces molecules thought to play anti-inflammatory roles in tissue remodeling and repair (*e.g*., IL-10 and transforming growth factor beta [TGF-β]) (131).

Notably, macrophages *in vivo* are exposed to a multitude of different stimuli, making them difficult to distinctly classify into M1 or M2. Studies quantifying M1 and M2 macrophages in AAA have not been entirely conclusive, with one study showing a higher proportion of M2 macrophages in the adventitia (30), and another demonstrating predominantly M1 macrophages in the adventitia with a higher proportion of M2 macrophages in the intraluminal thrombus (11). Therefore, most likely due to the complexity of human AAA wall tissue and the human organism, the current evidence suggests no clear polarization toward either macrophage phenotype.

Various proinflammatory cytokines secreted by M1 macrophages (*e.g*., TNF-α, IL-6, IL-1β, and IFN-γ) were found to be elevated in the serum of AAA patients (53). Murine animal models confirmed central roles for these cytokines in aortic disease, as deletion of TNF-α (150), IL-1β (52), and IFN-γ (151) resulted in reduced aneurysm formation, and deletion of IL-6 led to fewer aortic dissections (136).

Animal models have suggested a predominance of M1 macrophages in the AAA wall during early stage disease, whereas later-stage AAA seems to be associated with a shift toward M2 polarization. Infusion of male ApoE^−/−^ mice with AngII induces AAA formation and an increase in the M1/M2 ratio. This increase was detected as early as 7–10 days after starting the infusion and persisted until day 28 (105). A shift favoring the healing M2 rather than proinflammatory M1 phenotype in advanced AAA disease might counteract aneurysm growth and rupture. However, AngII infusion of ApoE^−/−^ mice for an additional 56 days both increased luminal diameters and aneurysmal rupture-associated deaths, and was associated with accumulation of macrophages that were consistent with an M2 phenotype (109).

Macrophages are involved in ECM degeneration, inflammation, and tissue healing and repair processes within the AAA wall. ECM degeneration is promoted by an increase of proteases such as cathepsins and matrix metalloproteinases (MMPs), and a decrease of their inhibitors (*e.g*., TIMP). Elastase-induced aneurysm formation in mice was suppressed by treatment with doxycycline as a nonselective MMP inhibitor (104). MMP-9-deficient mice were shown to be resistant to elastase-induced aneurysm formation, and they lost this resistance after bone marrow transplantation from wild-type animals (104). In support of this, TIMP-1 knockout mice showed a significant increase in elastase-induced aneurysm formation compared with wild-type animals (35).

The proinflammatory M1 macrophage phenotype seems to be more involved in ECM degeneration, expressing higher messenger RNA (mRNA) and protein levels of MMP-9 than M2 macrophages (11). ECM degeneration is thought to promote a self-sustaining inflammatory response, as certain breakdown products act as chemokines to further recruit monocytes (26). A repetitive peptide (Val-Gly-Val-Arg-Pro-Gly) found in human elastin is able to bind to cellular elastin receptors, promote monocyte chemotaxis to the AAA wall, and promote the inflammatory response that accompanies aneurysmal degeneration (Fig. 3) (45). Although ECM degeneration and inflammation are predominantly induced by M1 macrophages and M2 macrophages contain anti-inflammatory and tissue healing properties, recent studies suggest that the latter's nature is not entirely protective. For instance, it was demonstrated that CD163^+^ macrophages were associated with plaque progression, microvascularity, and expression of hypoxia-induced factor 1α (HIF1α) and vascular endothelial growth factor (VEGF)-A in human atherosclerotic lesions (44).

### Lymphocytes

Lymphocytes represent the majority of inflammatory cells within the AAA wall. Their mode of action is characterized by secretion of different proinflammatory cytokines, activation of various pathways promoting SMC apoptosis, and promoting synthesis of MMPs (31).

Lymphocytes are divided into B cells and T cells, with the latter are subdivided based on the expression of surface markers. Although modulatory T cells usually express CD4, most cytotoxic T cells express CD8 (26). Most inflammatory cells in AAA tissue are CD4^+^ T cells. Depending on stimulating factors, secretion products, and their functions, these are further subdivided into T helper (Th) or T effector (T_eff_) cells (*i.e*., Th1, Th2, Th17), and regulatory T cells (T_reg_) (26, 159).

Th1 cells are usually activated by IFN-γ or IL-12 (159). Activation of the “signal transducer and activator of transcription 4” (STAT4) and T-bet/TBX21 pathways results in secretion of IFN-γ, TNF-α, and IL-2 (159), which activate more Th1 cells, restrict polarization to other T cell subspecies, and activate macrophages (Table 1). Once activated, the macrophages produce IL-12, activating more Th1 cells (116). Thus, Th1 cells and macrophages show positive feedback stimulation, leading to ongoing augmentation of inflammation and ECM degeneration. Human AAA tissue expressed high mRNA levels of IFN-γ (in contrast to IL-4), suggesting a predominance of Th1 cells rather than Th2 cells. This hypothesis was fortified by an overexpression of the transcription factor T-bet, in the absence of GATA-3 expression (39).

Th1 cells and their secretion products seem to be associated with aneurysm growth, as increased IFN-γ serum levels correlated with AAA growth rate (53). Furthermore, deficiency of CD4^+^ T cells in a calcium chloride (CaCl_2_)-induced murine aneurysm model was shown to be related to lower expression of MMP and inhibition of aneurysm development. Replacement of IFN-γ by reinfusion of competent splenocytes from wild-type mice promoted aneurysm formation in these CD4^−/−^ animals (151), underlining the crucial role of IFN-γ in AAA disease.

Polarization toward the Th2 phenotype is promoted by IL-2 and IL-4. STAT6 and GATA-3 pathways lead to secretion of IL-4, IL-5, IL-10, IL-13, and IL-25 (Table 1) (159). Notably, IL-13 is an activator of anti-inflammatory M2 macrophages. The effects of these pathways on MMPs seem to be variable, as IL-4 suppressed collagenase expression in human monocytes (21), whereas IL-13 induced MMP-2, -9, -12, -13, and -14 (62).

At variance with the data suggesting predominance of Th1-specific cytokines (IFN-γ) and pathways (T-bet) in human AAA tissue (39), another study indicated a predominance of Th2-associated cytokines (IL-4, -5, -10), with Th1-characteristic cytokines (IL-2, -15) showing only low-level expression (116). The same research group showed that in allografted mouse aortas, IFN-γ deficiency promoted AAA development and increased levels of MMP-9 and -12. However, IL-4 deficiency seemed to protect against AAA formation (120), suggesting an imbalance toward the Th2 phenotype in AAA disease. The conflicting results regarding Th1/Th2 phenotype predominance might be explained by interspecies (murine AAA model *vs*. human AAA) differences, or changes at different stages of AAA disease (earlier phases in mouse model *vs*. advanced phases in human AAA).

Th17 lymphocytes are mainly stimulated by IL-1, IL-6, and IL-23, which activate the retinoic acid receptor-related orphan receptor γt (RORγt) and STAT3 pathways, resulting in secretion of IL-17 isoforms A and F, IL-21, and IL-22 (Table 1) (159). In a mouse model (AngII infusion in ApoE^−/−^ animals on high-fat diet), IL-17A was found to promote aortic superoxide production as well as aortic leukocyte and dendritic cell infiltration, but did not seem to affect aneurysm formation (79). However, in a different mouse model (elastase perfusion), knockout of IL-17 and IL-23 resulted in reductions in aneurysm diameter and cytokine levels (MCP-1, RANTES, KC, TNF-α, MIP-1α, and IFN-γ) (119). Furthermore, it has been demonstrated that human AAA tissue expresses significantly increased levels of IL-17 and IL-23 (119), suggesting that Th17 cells contribute to AAA disease.

The main role of T_reg_ cells seems to be to antagonize the aforementioned mainly proinflammatory T_eff_ cells. They are stimulated by IL-2 and TGF-β, activating STAT5 and forkhead box P3 (Foxp3) pathways, resulting in IL-10 and TGF-β secretion (Table 1) (159). T_reg_ cells have antiproliferative effects on T_eff_ cells (117). A loss of T_reg_ cells (relative to T_eff_ cells) may increase the proinflammatory milieu within the AAA wall. A relative reduction of T_reg_ cells compared with T_eff_ cells has been found in AAA tissue, with reduced levels of Foxp3 expression in peripheral CD4^+^CD25^+^ T_reg_ cells of AAA patients (152).

In summary, with respect to T lymphocyte involvement in AAA disease, the mechanism is thought to involve an imbalance within T_eff_ cells, which (despite conflicting data) seems to be in favor of the Th1 phenotype in humans, with minimization of T_reg_ cells. This disequilibrium promotes a proinflammatory environment with ECM degeneration leading to AAA progression.

Compared with T cells, the evidence on B lymphocytes and their involvement in AAA disease is sparse (156). B cells are categorized into B1 and B2 cells. After activation by T cells, B1 cells produce IgM antibodies. Activation of B2 cells can cause them to undergo isotype switching to become plasma cells, secreting large amounts of highly specific IgG antibodies. These, in turn, can activate the complement system, resulting in the activation of anaphylatoxins and the formation of the membrane attack complex (MAC) (156).

B lymphocytes have also been shown to be increased in the AAA wall, mainly in the adventitia (Fig. 4) (57). Indeed research suggests that, within the adventitia, the majority of lymphocytes are B cells (CD19^+^CD22^+^) (37). Atherosclerosis models have given rise to the concept of vascular-associated lymphoid tissue (VALT), consisting of disseminated accumulations of immunocompetent and antigen presenting cells (145). Whereas inflammatory cells in atherosclerosis typically accumulate within the arterial intima, VALT in AAA disease seems to accumulate in the adventitia, where it can organize into lymphoid follicles, aggregated in lymph node-like structures (9). Within these lymphoid follicles, B cells were found to form germinative centers (9, 156). The nodular centers also contained follicular dendritic cells, T lymphocytes, and macrophages (49).

The evidence regarding the effects of B cells on aneurysm growth is somewhat conflicting. One study showed that deficiency of B cells protected mice from developing elastase-induced AAAs, which was attributed to the absence of IgG-mediated complement activation (158). However, another study found that B cell-deficient mice were equally prone to AAA formation compared with wild-type mice. Adoptive transfer of B2 cells was even shown to suppress AAA formation, presumably due to an increase in splenic T_reg_-cells (84).

Autoantigens, such as aneurysm-associated protein-40 (AAAP-40) (149) or carbonic anhydrase 1 (CA1) (2), have been proposed to activate B cells. Owing to a cross reaction between antibodies against outer membrane proteins of Chlamydia pneumoniae and the heavy chain of immunoglobulins within the AAA wall, molecular mimicry has been proposed as an initiator of AAA formation after previous infection (77).

Activation of B2 lymphocytes leads to the secretion of IgG antibodies, of which IgG1, IgG2, and IgG3 were found to be increased in the AAA wall, as was the complement component C3. This observation was interpreted to suggest that IgG1, IgG2, and IgG3 may activate the complement system by the classical pathway, thereby promoting matrix proteolysis in AAA (14). This was supported by another study demonstrating upregulation of C1q and C4 in all AAA wall layers (3).

In the elastase-induced AAA mouse model, it was shown that IgG antibodies activate C3 convertase, which is a central enzyme of all three complement pathways. Deficiency in B cells was associated with abrogated C3 deposition in the elastase-perfused aortic wall and with protection from AAA formation (158).

Activation of C3 convertase leads to formation of MAC, and an important regulator of MAC (CD59) was shown to be downregulated in human AAA tissue (47). In the AngII-induced AAA mouse model, CD59 was shown to protect from AAA formation. Furthermore, it was demonstrated that MAC activates c-Jun and nuclear factor kappa-light-chain-enhancer of activated B cells (NF-κB) signaling pathways, which promote upregulation of MMP-2 and MMP-9 (148), presumably leading to degradation of ECM proteins.

The alternative complement pathway leads *via* C3 convertase to generation of C3a and C5a. In the elastase AAA mouse model, these were shown to recruit neutrophil leukocytes to the aortic wall promoting AAA formation (96).

B cells in the aortic wall produce pro- (IFN-γ, IL-6, and TNF-α) and anti-inflammatory cytokines (IL-2, IL-4, and IL-10) (84, 134). Results from a murine aneurysm model suggested that TNF-α promotes MMP-2 and MMP-9 expression, increasing macrophage infiltration into the aortic tissue, thereby leading to aneurysm formation (150).

In summary, despite a relative paucity of evidence, B lymphocytes appear to play an important role in the pathogenesis of AAA disease, especially by activating the complement cascade and by recruitment of other inflammatory cells, converging in inflammation and degradation of the ECM.

### Mast cells

Mast cells have also been found to be involved in AAA development. The number of mast cells is increased in the outer media and the adventitia of human AAA walls. Furthermore, mast cell-deficient mutant rats were resistant to CaCl_2_-induced aortic aneurysm. In a cell culture experiment, it was shown that mast cells directly augmented the activity of MMP-9 produced by monocytes or macrophages (137). In addition, chymases (serine proteases exclusively secreted by mast cells) were found to induce SMC apoptosis (69) and to be involved in activation of promatrix metalloprotease 9 (pro-MMP-9) and pro-MMP-2 (135). However, inhibition of mast cells in humans did not alter AAA growth in a randomized controlled trial during 12-month follow-up (121). Although mast cell inhibition does not seem to affect the growth rate of a fully evolved AAA, a possibly beneficial effect in the early stages of AAA formation needs further investigation.

### Neutrophil leukocytes

Neutrophils produce a variety of proteases and collagenases (1), which supposedly are involved in ECM degradation and ultimately in aneurysm rupture (29). Neutrophils are also thought to promote AAA formation *via* mechanisms independent of MMPs (33). In the elastase-induced AAA mouse model, neutrophil infiltration was observed in the AAA wall. If treated with antineutrophil antibody (resulting in neutropenia before elastase perfusion), mice were protected from AAA formation, but without alteration in MMPs (33).

Formation of neutrophil extracellular traps (NETs), mainly to entrap pathogens, is a defense mechanism enacted by neutrophils. NETosis, which was demonstrated to be promoted by IL-1β, seems to play a role in AAA formation, and inhibition of NETosis significantly attenuated AAA formation in a mouse model (85).

Neutrophils are also known to possess pro-oxidant activities *via* nicotinamide adenine dinucleotide phosphate (NADPH) oxidase and myeloperoxidase, producing reactive oxygen species and reactive nitrogen species. Circulating polymorphonuclear neutrophils from AAA patients were found to contain higher H_2_O_2_ and myeloperoxidase levels, and diminished catalase levels compared with control patients, underlining a relevant role for oxidative stress in AAA disease (107).

IL-8, which is an important chemoattractant cytokine for neutrophils, was upregulated 11-fold in the AAA wall compared with controls (88), Another study found that neutrophil-derived leukotriene B (4), which is a major neutrophil chemotactic factor, is released from the intraluminal thrombus (48), supporting the finding that most leukocytes are found at the luminal layer of the intraluminal thrombus (101).

Taken together, the available evidence suggests that neutrophils participate in AAA development through the promotion of oxidative stress, secretion of proteolytic agents, and NET formation.

## The Role of ncRNA in AAA

Sequencing of the human genome has shown that only ∼1.5% contains protein-coding sequences. Together with introns within protein-coding genes and 5′- and 3′-untranslated regions, the combination occupies ∼28% of the human genome (38).

However, ∼80% of the genome potentially participates to some extent in biochemical activities. Despite not being translated into proteins, ∼70% of the genome (at least) is transcribed into mRNA (20). These elements are referred to as ncRNA.

There exist constitutive types of housekeeping ncRNAs, which are expressed in all cells and possess well-defined functions within the cell. This class includes transfer RNAs (tRNAs), ribosomal RNAs (rRNA), small nucleolus RNAs (snoRNAs), small nuclear RNAs (snRNAs), and possibly also telomere complex-associated guide RNAs (38).

Beyond this, there are regulatory ncRNAs, which can be expressed in a highly regulated manner in different cell types and/or during different developmental periods. Owing to their various functions in different physiologic and pathologic processes, these have attracted substantial attention within the past decade. ncRNAs are usually classified based on their size, with short noncoding RNAs (miRNAs) and long noncoding RNAs (lncRNAs) being defined as smaller or larger than 200 nucleotides, respectively.

### Short noncoding RNA

**FIG. 5.**
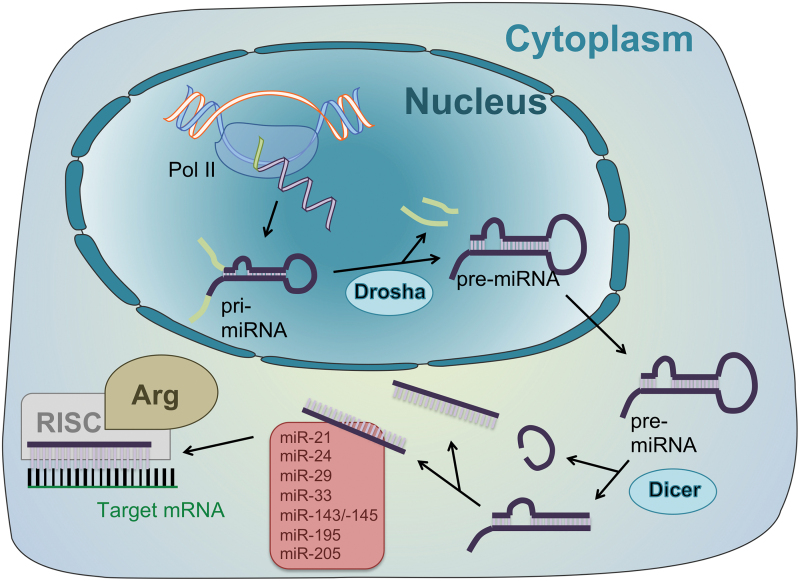
**Processing and mode of action of miRNAs.** Once transcribed by Pol II, an RNase named Drosha processes pri-miRNA into pre-miRNA. The latter is transported into the cytoplasm, where processing mediated by another RNase called Dicer leads to formation of mature miRNA. Together with argonaute, the different miRNAs incorporate into RISCs, which bind to distinct mRNAs and thereby suppress translation into various proteins. Arg, argonaute; miRNA, short noncoding ribonucleic acid; mRNA, messenger RNA; Pol II, RNA polymerase II; RISC, RNA-induced silencing complex. Color images are available online.

miRNAs are short (∼18 to 23 nucleotides) single-stranded RNAs (6, 60). They are transcribed as long primary transcripts (pri-miRNAs), which are processed in the nucleus into stem-loop precursors of ∼70 nucleotides (pre-miRNAs). This step is mediated by an RNase III called Drosha. After active transportation into the cytoplasm, pre-miRNA is processed into mature miRNAs, mediated by another member of the RNase III family named Dicer (67). Please refer to Figure 5 for a schematic illustration of miRNA processing and mode of action. Mature miRNAs are involved in post-transcriptional processes, and commonly repress the expression of target genes by incorporating together with argonaute into a protein complex called the RNA-induced silencing complex (RISC) and binding to the 3’ untranslated region of mRNA (6). Accordingly, one miRNA can bind to and potentially suppress multiple mRNAs (60).

Contributions from miRNAs to inflammatory processes have been demonstrated for a multitude of diseases (123), including rheumatoid arthritis (126), psoriasis (124), asthma (78), ulcerative colitis (147), systemic lupus erythematosus (133), different forms of glomerulonephritis (93), and also atherosclerosis (51, 127).

Furthermore, various miRNAs have been established to be involved in the cell fates of vascular SMCs and in AAA (Table 2) (60, 68, 74). However, the evidence on miRNA species involvement in the inflammatory processes driving AAA disease is limited.

**Table 2.** Involvement of Short NonCoding RNAs in Abdominal Aortic Aneurysm Disease and Underlying Inflammatory Processes

Shading indicates involvement in inflammatory processes.

AAA, abdominal aortic aneurysm; ABCA1, adenosine triphosphate-binding cassette transporter A1; Chi3l1, chitinase 3-like 1; EC, endothelial cell; ECM, extracellular matrix; FB, fibroblast; MAPK, mitogen-activated protein kinase; miRNA, short noncoding RNA; MPh, macrophage; PTEN, phosphatase and tensin homolog; SMC, smooth muscle cell; TIMP, tissue inhibitor of metalloproteinases.

**FIG. 6.**
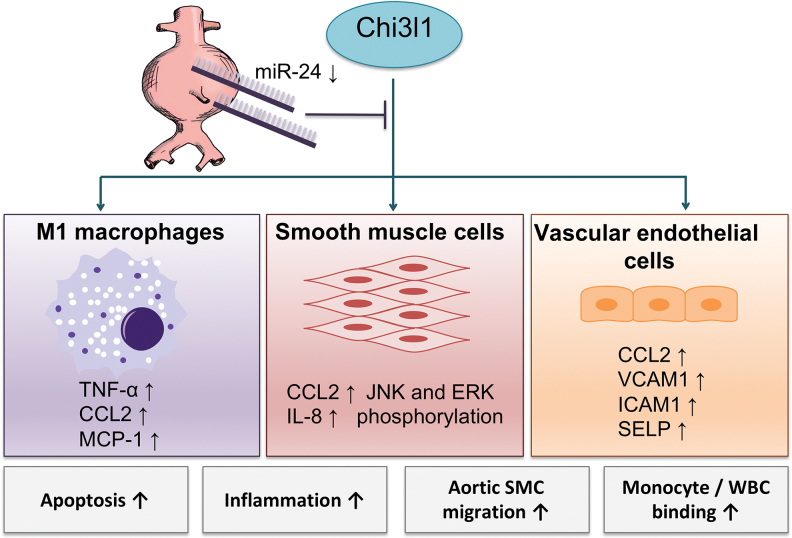
**Different effects of miR-24 in AAA disease.** The expression of miR-24 is decreased in AAA, leading to an increased expression of Chi3l1. This again promotes the synthesis of various cytokines in M1 macrophages, SMCs, and vascular endothelial cells, converging toward proapoptotic and proinflammatory processes (82). Chi3l1, chitinase 3-like 1; WBC, white blood cell. Color images are available online.

An association with those inflammatory processes was demonstrated for miR-24, seemingly pertaining to its regulation of chitinase 3-like 1 (Chi3l1). By reducing the expression of Chi3l1, miR-24 inhibits cytokine synthesis (*e.g*., IL-8 and CCL2) in macrophages and their survival, and thereby limits inflammation and ECM degeneration (Fig. 6) (82). Accordingly, overexpression of miR-24 inhibited AAA growth in a mouse model (82). In human macrophages, overexpression of miR-24 attenuated phagocytosis and secretion of inflammatory cytokines (*e.g*., TNF-α, IL-6, and IL-12p40) (94). Similar effects were demonstrated for miR-30b and miR-142-3p (94), although for these, a relation with AAA disease has not been established yet.

Another miRNA, miR-33, affected AAA formation through monocyte and macrophage regulation. Knockout of miR-33 was associated with less accumulation of macrophages, and lower expression of monocyte chemotactic protein-1 within the aortic wall. In addition, peritoneal macrophages from miR-33^−/−^ mice were found to express lesser levels of MMP-9 (92). MiR-33 knockout mice showed decreased AAA formation after either AngII or CaCl_2_ treatment (92). Apart from these proinflammatory functions, miR-33 is also involved in cholesterol homeostasis. In human and murine cells, miR-33 inhibits the expression of the ABCA1, limiting cholesterol efflux to apolipoprotein A1 and reducing circulating high-density lipoprotein levels (Table 2) (110).

Furthermore, miR-155 has been found to enhance vascular inflammation by suppressing BCL6 expression, which itself attenuates proinflammatory NF-κB signaling (95). The proinflammatory properties of miR-155 may be attributable to its effects on macrophage activation and polarization (25), with increased miR-155 levels in M1 macrophages and decreased levels in the M2 phenotype (12). Notably, apart from elevated expression in M1 macrophages, miR-155 also supports macrophage polarization toward the proinflammatory M1 phenotype. Similar effects were demonstrated for miR-125b (17) and miR-127 (153), which equally have not been implicated in AAA disease yet.

This also applies to miR-342-5p, which alongside miR-155 was found to be upregulated in early atherosclerotic lesions in ApoE^−/−^ mice. By suppression of Akt1, miR-342-5p causes an upregulation of miR-155, which again induces proinflammatory mediators such as Nos2 and IL6 in macrophages (143). Therefore, a synergy of miR-342-5p and miR-155 is supposed to drive macrophages toward a proinflammatory and proatherogenic state (144).

Although a possible involvement in AAA disease still is to be demonstrated, miR-103 was shown to play a role in atherosclerosis by promoting inflammation and endoplasmatic reticulum stress in endothelial cells derived from a mouse model (51). Depletion of miR-103 was shown to counteract atherosclerosis through blocking phosphatase and tensin homolog (PTEN)-mediated mitogen-activated protein kinase (MAPK) signaling (51).

In contrast, miR-181a seems to suppress inflammation by decreasing proinflammatory gene expression (*e.g*., VCAM-1, ICAM-1, and E-selectin) and infiltration of macrophages, leukocytes, and T cells into atherosclerotic plaques (127). Again, no direct association with AAA disease was shown so far.

Notably, the predominating mechanisms of action of most miRNAs that are known to be involved in AAA disease are not directly related to myeloid cells and inflammation. Overexpression of miR-21 was seen in human AAA samples as well as mouse models of AAA (80). Lentiviral overexpression of miR-21 decreased the expression of the PTEN protein, and promoting downstream activation of the serine–threonine kinase AKT, which itself has proproliferative and antiapoptotic properties (Table 2). Overexpression of miR-21 inhibited AAA growth, whereas inhibition of miR-21 promoted AAA expansion (80). miR-21 has further been evaluated for its role in vascular inflammation and myeloid cell activation in the context of atherosclerosis. Canfran-Duque *et al.* found that lowering miR-21 in macrophages accelerates atherosclerosis and plaque necrosis by increasing the expression of MKK3, an upstream mediator of p38-CHOP and JNK signaling (13).

In contrast, miR-29 promotes AAA formation by inhibition of expression of ECM proteins, including collagens (COL1A1, COL3A1, and COL5A1) and elastin (Table 2) (81). In murine animal models, overexpression of miR-29b resulted in augmented AAA growth and a significantly higher aortic rupture rate. Conversely, inhibition of miR-29b by administration of locked nucleic acid anti-miR-29b reduced AAA progression by increasing collagen expression, which seemed to stabilize the aortic wall (81). Also, inhibition of miR-29 was found to decrease the expression of MMP-9 in the aorta (10).

Upregulation of miR-29b with advanced age was shown in mice, suggesting involvement in older patients' susceptibility to AAAs (10). Furthermore, elevated miR-29b levels were found in human thoracic aortic aneurysms (10) and in the aortic roots of Marfan (Fbn1^C1039G/+^) mice (87). In the latter, increased miR-29b expression was shown to result from a decreased activation of NF-κB, which seemed to act as a repressor (87).

Another miRNA that affects aortic ECM remodeling is miR-195, which targets collagens, elastin, and MMPs (154). Plasma levels of miR-195 showed an inverse correlation with human AAA diameter.

The miR-143/145 cluster is involved in SMC differentiation, inducing a contractile, quiescent, and mature phenotype (32), potentially stabilizing the AAA wall (60). This is achieved by inhibition of multiple factors, including Elk-1, Klf4, and CamkII-δ, and activation of Myocd (22). The expression of miR-143 and -145 is decreased in human AAA tissue compared with undiseased aortas. A loss of miR-143 and miR-145 expression seems to be associated with incomplete differentiation of SMCs and alteration of the aortic wall, promoting AAA formation (32).

In summary, despite growing evidence on the involvement of miRNAs in AAA disease, their effect on myeloid cell function and inflammation is still understudied.

### Long noncoding RNA

As noted, ncRNA fragments exceeding a length of 200 nucleotides are referred to as lncRNAs. Acting as signaling cues, decoys, scaffolds, or miRNA sponges, their modes of action are manifold and generally different from those described for miRNAs (*i.e*., complementary binding) (60).

In relation to miRNAs, involvement in various inflammatory conditions including rheumatoid arthritis (125), osteoarthritis (99), celiac disease (15), multiple sclerosis (155), and Kawasaki disease (75) was demonstrated (18, 83). However, their role in AAA disease and particularly in the inflammatory processes underlying AAA disease is much less investigated.

**FIG. 7.**
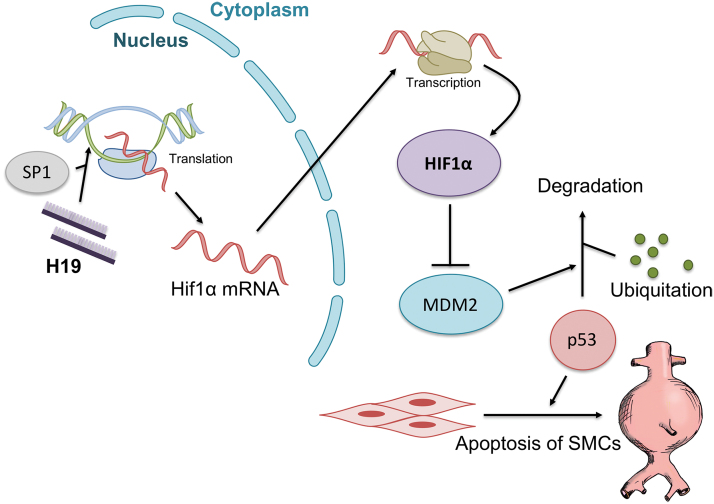
**Schematic effect of H19 on vascular SMC apoptosis in AAA disease.** H19 induces AAA progression by promoting Hif1α translation in the nucleus under the influence of SP1 recruitment. In the cytoplasm, HIF1α protein is transcribed and inhibits MDM2. This results in reduced degradation of p53, leading to SMC apoptosis and AAA formation (71). HIF1α, hypoxia-induced factor 1α; MDM2, murine double minute 2; SP1, specificity protein 1. Color images are available online.

So far, H19 is the only lncRNA found to be involved in AAA formation (71). Although involvement in vascular disease has been demonstrated for a number of other lncRNAs (5, 70, 73), distinctive functional roles in AAA formation have not been proven. Knockdown of H19 using antisense oligonucleotides led to a significant reduction of aneurysm formation in two different murine AAA models. Upregulation of H19 promoted SMC apoptosis in the aneurysm wall (Fig. 7). Cultured human SMCs showed decreased apoptotic rates after knockdown of H19, and in this context apoptosis of SMCs seems to be mediated by the transcription factor HIF1α (71).

To date, there is no evidence on the contribution of lncRNAs to inflammatory processes underlying AAA disease.

## Conclusions and Future Directions

Myeloid cells are involved in AAA development and growth. Over the past decades, a large body of evidence has accumulated on monocytes, macrophages, and lymphocytes and their contribution to AAA. In contrast, knowledge on the role of neutrophils and mast cells with respect to AAA disease is still minimal. In summary, the early phase of AAA development seems to be characterized by an imbalance of myeloid cells toward proinflammatory cell types and phenotypes (39, 53, 105). M1 macrophages from different origins accumulate within the aortic wall (34), whereas lymphocytes (especially B cells) predominantly aggregate within the adventitia (57). Interaction of M1 macrophages and Th1 lymphocytes induces secretion of different proinflammatory and ECM-degrading cytokines (*e.g*., TNF-α, IFN-γ, IL-6, MMPs, and iNOS) (131, 159), which in conjunction with activation of the complement cascade by B cells (3, 14) sustains a cytotoxic and ECM-degrading environment. ECM degradation products themselves contribute to this milieu by recruiting additional monocytes (26). Evidence is growing that mechanisms involving ncRNAs contribute by altering this milieu (82, 92, 95, 110).

Understanding the distinct mechanisms of AAA disease is considered a prerequisite to reach two high-priority targets. First, factors shown to be involved in the development of AAA might eventually serve as biomarkers to identify patients at risk for later AAA formation and to predict the course of disease. Regarding myeloid cells, changes in circulating monocyte subsets (40) or variations in gene or protein expression (115) potentially might serve as predictors for AAA growth. Diminished catalase levels in circulating polymorphonuclear neutrophils and plasma were found to be associated with the presence of AAA (107). Markers of ECM degeneration including elastin peptides and MMP-9, and inflammatory markers such as IFN-γ (53) and MIF (97) have been proposed as biomarkers for AAA disease (46). With respect to ncRNAs, miR-24 may represent a biomarker for AAA development (60), and miR-195 might be of prognostic value to predict the growth of AAAs (141).

The second aim of understanding the mechanisms of AAA disease is to discover possible targets for medical intervention at earlystage. Such medical intervention might counteract AAA development or stabilize the aortic wall, and thereby prevent future AAA growth and rupture. Inhibition of MCP-1 (specifically in bone marrow-derived cells), to reduce monocyte recruitment into the aortic wall, has been proposed as a potential therapeutic option (89). Inducing a shift of macrophages from a proinflammatory M1 phenotype toward an anti-inflammatory M2 phenotype might also be effective. A promising approach to attenuate AAA formation is the inhibition of AAA enhancing miRNAs. For instance, the inhibition of miR-29b (using anti-miRs) reduced AAA progression in a mouse model (81).

An alternative therapeutic option might be the overexpression or local delivery of AAA-attenuating miRNAs. Possible targets could include miR-21 or miR-24. Owing to pharmacological challenges (*e.g*., degradation in serum by nucleases or endocytic escape) and difficulties with local delivery, systemic or local administration of naked miRNA mimics or miRNA mimics encoded in viral vectors has proven ineffective in other contexts (113). Chemical modifications (*e.g*., methylation or locked nucleic acids) or the use of delivery systems (*e.g*., lipid nanoparticles) are possible options to overcome these issues (113). Another problem to be solved is potential toxicity related to off-target effects.

Although there is a decent body of evidence to investigate potential effects of anti-inflammatory substances on AAA evolvement in animal models, the literature on AAA attenuating drugs in humans is limited (41, 58). Interestingly, one singular study assessed the effect of nonsteroidal anti-inflammatory drugs (NSAIDs) on AAA development. Only published as a conference abstract, the authors report a reduction in AAA growth rate (1.8 *vs*. 3.2 mm/year) in patients taking NSAIDs (*n* = 19) compared with a matched control group (*n* = 59) (36). To date, only one randomized controlled trial to assess a potential effect of an anti-inflammatory substance in human was performed. In the AORTA trial, AAA patients were allocated to different doses of the mast cell inhibitor pemirolast or placebo. After a follow-up of 12 months, no significant difference was seen in AAA growth rates in between all study arms (121). However, as the study was performed on patients who already had fully evolved AAAs, these results cannot preclude a potential effect of mast cell inhibition on early stage disease.

A potential beneficial effect of anti-inflammatory substances on AAA formation in the early stages of disease is underpinned by a recent study to investigate the impact of anti-inflammatory diet on AAA incidence. In 81,705 patients, the anti-inflammatory diet index (AIDI) was inversely associated with both ruptured and nonruptured AAA incidences (54).

One major issue in the field of AAA basic research is that most current evidence on myeloid cells and ncRNAs originates from small animal models, which makes extrapolation of results difficult. Although it is feasible to collect human AAA samples from OSR patients, acquiring samples is becoming more challenging as a growing proportion of AAA patients are treated with endovascular techniques. In addition, human AAA tissue samples nearly always reflect end-stage disease, leaving the critical mechanisms behind AAA initiation undetected.

The use of large animal models might play an important role to reduce interspecies variations and to make results more applicable to humans. Earlier studies have already been performed, showing the contribution of the lncRNA H19 during AAA formation in a porcine pancreatic elastase-induced Yucatan Ldlr^−/−^ (low-density lipoprotein receptor) mini-pig aneurysm model (71). Further research using this model will potentially pave the way for novel therapeutics that inhibit aneurysm growth and limit the risk of fatal acute ruptures.

## Abbreviations Used

AAA: abdominal aortic aneurysm
ABCA1: adenosine triphosphate-binding cassette transporter A1
AngII: angiotensin II
ApoE: apolipoprotein E
CaCl_2_: calcium chloride
Chi3l1: chitinase 3-like 1
CX_3_CR1: C-X_3_-C motif chemokine receptor 1
ECM: extracellular matrix
EVAR: endovascular aortic repair
Foxp3: forkhead box P3
HIF1α: hypoxia-induced factor 1α
IFN-γ: interferon gamma
lncRNAs: long noncoding RNA
LPS: lipopolysaccharide
MAC: membrane attack complex
miRNA: short noncoding RNA
MMPs: matrix metalloproteinases
mRNA: messenger RNA
ncRNA: noncoding RNA
NET: neutrophil extracellular trap
NF-κB: nuclear factor kappa-light-chain-enhancer of activated B cells
NSAID: nonsteroidal anti-inflammatory drug
OSR: open surgical repair
PAOD: peripheral arterial occlusive disease
PTEN: phosphatase and tensin homolog
SMC: smooth muscle cell
STAT: signal transducer and activator of transcription
T_eff_: T effector
TGF-β: transforming growth factor beta
TIMP: tissue inhibitor of metalloproteinases
TNF-α: tumor necrosis factor alpha
T_reg_: regulatory T
VALT: vascular-associated lymphoid tissue

## References

[1] Abdul-Hussien H, Soekhoe RG, Weber E, von der Thusen JH, Kleemann R, Mulder A, van Bockel JH, Hanemaaijer R, and Lindeman JH Collagen degradation in the abdominal aneurysm: a conspiracy of matrix metalloproteinase and cysteine collagenases. Am J Pathol 170: 809–817, 2007 17322367 10.2353/ajpath.2007.060522 PMC1864891

[2] Ando T, Iizuka N, Sato T, Chikada M, Kurokawa MS, Arito M, Okamoto K, Suematsu N, Makuuchi H, and Kato T Autoantigenicity of carbonic anhydrase 1 in patients with abdominal aortic aneurysm, revealed by proteomic surveillance. Hum Immunol 74: 852–857, 2013 23557951 10.1016/j.humimm.2013.02.009

[3] Ando T, Nagai K, Chikada M, Okamoto K, Kurokawa M, Kobayashi T, Kato T, and Makuuchi H Proteomic analyses of aortic wall in patients with abdominal aortic aneurysm. J Cardiovasc Surg (Torino) 52: 545–555, 2011 21792162

[4] Bahia SS, Vidal-Diez A, Seshasai SR, Shpitser I, Brownrigg JR, Patterson BO, Ray KK, Holt PJ, Thompson MM, and Karthikesalingam A Cardiovascular risk prevention and all-cause mortality in primary care patients with an abdominal aortic aneurysm. Br J Surg 103: 1626–1633, 2016 27704527 10.1002/bjs.10269

[5] Ballantyne MD, Pinel K, Dakin R, Vesey AT, Diver L, Mackenzie R, Garcia R, Welsh P, Sattar N, Hamilton G, Joshi N, Dweck MR, Miano JM, McBride MW, Newby DE, McDonald RA, and Baker AH Smooth muscle enriched long noncoding RNA (SMILR) regulates cell proliferation. Circulation 133: 2050–2065, 2016 27052414 10.1161/CIRCULATIONAHA.115.021019 PMC4872641

[6] Bartel DP. MicroRNAs: target recognition and regulatory functions. Cell 136: 215–233, 2009 19167326 10.1016/j.cell.2009.01.002 PMC3794896

[7] Belge KU, Dayyani F, Horelt A, Siedlar M, Frankenberger M, Frankenberger B, Espevik T, and Ziegler-Heitbrock L The proinflammatory CD14^+^CD16^+^DR^++^ monocytes are a major source of TNF. J Immunol 168: 3536–3542, 2002 11907116 10.4049/jimmunol.168.7.3536

[8] Blomkalns AL, Gavrila D, Thomas M, Neltner BS, Blanco VM, Benjamin SB, McCormick ML, Stoll LL, Denning GM, Collins SP, Qin Z, Daugherty A, Cassis LA, Thompson RW, Weiss RM, Lindower PD, Pinney SM, Chatterjee T, and Weintraub NL CD14 directs adventitial macrophage precursor recruitment: role in early abdominal aortic aneurysm formation. J Am Heart Assoc 2: e000065, 2013 23537804 10.1161/JAHA.112.000065 PMC3647288

[9] Bobryshev YV and Lord RS Vascular-associated lymphoid tissue (VALT) involvement in aortic aneurysm. Atherosclerosis 154: 15–21, 2001 11137078 10.1016/s0021-9150(00)00441-x

[10] Boon RA, Seeger T, Heydt S, Fischer A, Hergenreider E, Horrevoets AJ, Vinciguerra M, Rosenthal N, Sciacca S, Pilato M, van Heijningen P, Essers J, Brandes RP, Zeiher AM, and Dimmeler S MicroRNA-29 in aortic dilation: implications for aneurysm formation. Circ Res 109: 1115–1119, 2011 21903938 10.1161/CIRCRESAHA.111.255737

[11] Boytard L, Spear R, Chinetti-Gbaguidi G, Acosta-Martin AE, Vanhoutte J, Lamblin N, Staels B, Amouyel P, Haulon S, and Pinet F Role of proinflammatory CD68(+) mannose receptor(−) macrophages in peroxiredoxin-1 expression and in abdominal aortic aneurysms in humans. Arterioscler Thromb Vasc Biol 33: 431–438, 2013 23241402 10.1161/ATVBAHA.112.300663

[12] Cai X, Yin Y, Li N, Zhu D, Zhang J, Zhang CY, and Zen K Re-polarization of tumor-associated macrophages to pro-inflammatory M1 macrophages by microRNA-155. J Mol Cell Biol 4: 341–343, 2012 22831835 10.1093/jmcb/mjs044

[13] Canfran-Duque A, Rotllan N, Zhang X, Fernandez-Fuertes M, Ramirez-Hidalgo C, Araldi E, Daimiel L, Busto R, Fernandez-Hernando C, and Suarez Y Macrophage deficiency of miR-21 promotes apoptosis, plaque necrosis, and vascular inflammation during atherogenesis. EMBO Mol Med 9: 1244–1262, 2017 28674080 10.15252/emmm.201607492 PMC5582411

[14] Capella JF, Paik DC, Yin NX, Gervasoni JE, and Tilson MD Complement activation and subclassification of tissue immunoglobulin G in the abdominal aortic aneurysm. J Surg Res 65: 31–33, 1996 8895603 10.1006/jsre.1996.0339

[15] Castellanos-Rubio A, Fernandez-Jimenez N, Kratchmarov R, Luo X, Bhagat G, Green PH, Schneider R, Kiledjian M, Bilbao JR, and Ghosh S A long noncoding RNA associated with susceptibility to celiac disease. Science 352: 91–95, 2016 27034373 10.1126/science.aad0467 PMC4994711

[16] Chaikof EL, Dalman RL, Eskandari MK, Jackson BM, Lee WA, Mansour MA, Mastracci TM, Mell M, Murad MH, Nguyen LL, Oderich GS, Patel MS, Schermerhorn ML, and Starnes BW The Society for Vascular Surgery practice guidelines on the care of patients with an abdominal aortic aneurysm. J Vasc Surg 67: 2.e2–77.e2, 2018 10.1016/j.jvs.2017.10.044 29268916

[17] Chaudhuri AA, So AY, Sinha N, Gibson WS, Taganov KD, O'Connell RM, and Baltimore D MicroRNA-125b potentiates macrophage activation. J Immunol 187: 5062–5068, 2011 22003200 10.4049/jimmunol.1102001 PMC3208133

[18] Chen J, Ao L, and Yang J Long non-coding RNAs in diseases related to inflammation and immunity. Ann Transl Med 7: 494, 2019 31700930 10.21037/atm.2019.08.37 PMC6803193

[19] Combadiere C, Potteaux S, Rodero M, Simon T, Pezard A, Esposito B, Merval R, Proudfoot A, Tedgui A, and Mallat Z Combined inhibition of CCL2, CX_3_CR1, and CCR5 abrogates Ly6C(hi) and Ly6C(lo) monocytosis and almost abolishes atherosclerosis in hypercholesterolemic mice. Circulation 117: 1649–1657, 2008 18347211 10.1161/CIRCULATIONAHA.107.745091

[20] Consortium EP. An integrated encyclopedia of DNA elements in the human genome. Nature 489: 57–74, 2012 22955616 10.1038/nature11247 PMC3439153

[21] Corcoran ML, Stetler-Stevenson WG, Brown PD, and Wahl LM Interleukin 4 inhibition of prostaglandin E2 synthesis blocks interstitial collagenase and 92-kDa type IV collagenase/gelatinase production by human monocytes. J Biol Chem 267: 515–519, 1992 1309751

[22] Cordes KR, Sheehy NT, White MP, Berry EC, Morton SU, Muth AN, Lee TH, Miano JM, Ivey KN, and Srivastava D miR-145 and miR-143 regulate smooth muscle cell fate and plasticity. Nature 460: 705–710, 2009 19578358 10.1038/nature08195 PMC2769203

[23] Cosford PA and Leng GC Screening for abdominal aortic aneurysm. Cochrane Database Syst Rev 2: CD002945, 2007 10.1002/14651858.CD002945.pub2 17443519

[24] Cros J, Cagnard N, Woollard K, Patey N, Zhang SY, Senechal B, Puel A, Biswas SK, Moshous D, Picard C, Jais JP, D'Cruz D, Casanova JL, Trouillet C, and Geissmann F Human CD14dim monocytes patrol and sense nucleic acids and viruses via TLR7 and TLR8 receptors. Immunity 33: 375–386, 2010 20832340 10.1016/j.immuni.2010.08.012 PMC3063338

[25] Curtale G, Rubino M, and Locati M MicroRNAs as molecular switches in macrophage activation. Front Immunol 10: 799, 2019 31057539 10.3389/fimmu.2019.00799 PMC6478758

[26] Dale MA, Ruhlman MK, and Baxter BT Inflammatory cell phenotypes in AAAs: their role and potential as targets for therapy. Arterioscler Thromb Vasc Biol 35: 1746–1755, 2015 26044582 10.1161/ATVBAHA.115.305269 PMC4514552

[27] Danielson LS, Menendez S, Attolini CS, Guijarro MV, Bisogna M, Wei J, Socci ND, Levine DA, Michor F, and Hernando E A differentiation-based microRNA signature identifies leiomyosarcoma as a mesenchymal stem cell-related malignancy. Am J Pathol 177: 908–917, 2010 20558575 10.2353/ajpath.2010.091150 PMC2913343

[28] Davis BN, Hilyard AC, Nguyen PH, Lagna G, and Hata A Induction of microRNA-221 by platelet-derived growth factor signaling is critical for modulation of vascular smooth muscle phenotype. J Biol Chem 284: 3728–3738, 2009 19088079 10.1074/jbc.M808788200 PMC2635044

[29] Defawe OD, Colige A, Lambert CA, Delvenne P, Lapiere Ch M, Limet R, Nusgens BV, and Sakalihasan N Gradient of proteolytic enzymes, their inhibitors and matrix proteins expression in a ruptured abdominal aortic aneurysm. Eur J Clin Invest 34: 513–514, 2004 15255789 10.1111/j.1365-2362.2004.01371.x

[30] Dutertre CA, Clement M, Morvan M, Schakel K, Castier Y, Alsac JM, Michel JB, and Nicoletti A Deciphering the stromal and hematopoietic cell network of the adventitia from non-aneurysmal and aneurysmal human aorta. PLoS One 9: e89983, 2014 24587165 10.1371/journal.pone.0089983 PMC3937418

[31] Eagleton MJ. Inflammation in abdominal aortic aneurysms: cellular infiltrate and cytokine profiles. Vascular 20: 278–283, 2012 23091264 10.1258/vasc.2011.201207

[32] Elia L, Quintavalle M, Zhang J, Contu R, Cossu L, Latronico MV, Peterson KL, Indolfi C, Catalucci D, Chen J, Courtneidge SA, and Condorelli G The knockout of miR-143 and -145 alters smooth muscle cell maintenance and vascular homeostasis in mice: correlates with human disease. Cell Death Differ 16: 1590–1598, 2009 19816508 10.1038/cdd.2009.153 PMC3014107

[33] Eliason JL, Hannawa KK, Ailawadi G, Sinha I, Ford JW, Deogracias MP, Roelofs KJ, Woodrum DT, Ennis TL, Henke PK, Stanley JC, Thompson RW, and Upchurch GR Jr Neutrophil depletion inhibits experimental abdominal aortic aneurysm formation. Circulation 112: 232–240, 2005 16009808 10.1161/CIRCULATIONAHA.104.517391

[34] Ensan S, Li A, Besla R, Degousee N, Cosme J, Roufaiel M, Shikatani EA, El-Maklizi M, Williams JW, Robins L, Li C, Lewis B, Yun TJ, Lee JS, Wieghofer P, Khattar R, Farrokhi K, Byrne J, Ouzounian M, Zavitz CC, Levy GA, Bauer CM, Libby P, Husain M, Swirski FK, Cheong C, Prinz M, Hilgendorf I, Randolph GJ, Epelman S, Gramolini AO, Cybulsky MI, Rubin BB, and Robbins CS Self-renewing resident arterial macrophages arise from embryonic CX_3_CR1(+) precursors and circulating monocytes immediately after birth. Nat Immunol 17: 159–168, 2016 26642357 10.1038/ni.3343

[35] Eskandari MK, Vijungco JD, Flores A, Borensztajn J, Shively V, and Pearce WH Enhanced abdominal aortic aneurysm in TIMP-1-deficient mice. J Surg Res 123: 289–293, 2005 15680392 10.1016/j.jss.2004.07.247

[36] Franklin IJ, Walton LJ, Brown L, Greenhalgh RN, and Powell JT Vascular surgical society of Great Britain and Ireland: non-steroidal anti-inflammatory drugs to treat abdominal aortic aneurysm. Br J Surg 86: 707, 1999 10.1046/j.1365-2168.1999.0707b.x 10361345

[37] Freestone T, Turner RJ, Coady A, Higman DJ, Greenhalgh RM, and Powell JT Inflammation and matrix metalloproteinases in the enlarging abdominal aortic aneurysm. Arterioscler Thromb Vasc Biol 15: 1145–1151, 1995 7627708 10.1161/01.atv.15.8.1145

[38] Fu XD. Non-coding RNA: a new frontier in regulatory biology. Natl Sci Rev 1: 190–204, 2014 25821635 10.1093/nsr/nwu008 PMC4374487

[39] Galle C, Schandene L, Stordeur P, Peignois Y, Ferreira J, Wautrecht JC, Dereume JP, and Goldman M Predominance of type 1 CD4^+^ T cells in human abdominal aortic aneurysm. Clin Exp Immunol 142: 519–527, 2005 16297165 10.1111/j.1365-2249.2005.02938.x PMC1809544

[40] Ghigliotti G, Barisione C, Garibaldi S, Brunelli C, Palmieri D, Spinella G, Pane B, Spallarossa P, Altieri P, Fabbi P, Sambuceti G, and Palombo D CD16(+) monocyte subsets are increased in large abdominal aortic aneurysms and are differentially related with circulating and cell-associated biochemical and inflammatory biomarkers. Dis Markers 34: 131–142, 2013 23348634 10.3233/DMA-120956 PMC3809748

[41] Golledge J, Moxon JV, Singh TP, Bown MJ, Mani K, and Wanhainen A. Lack of an effective drug therapy for abdominal aortic aneurysm. J Intern Med 2019. [Epub ahead of print]; DOI:10.1111/joim.12958 31278799

[42] Gomez D and Owens GK Smooth muscle cell phenotypic switching in atherosclerosis. Cardiovasc Res 95: 156–164, 2012 22406749 10.1093/cvr/cvs115 PMC3388816

[43] Greenhalgh RM, Brown LC, Kwong GP, Powell JT, and Thompson SG; EVAR Trial Participants Comparison of endovascular aneurysm repair with open repair in patients with abdominal aortic aneurysm (EVAR trial 1), 30-day operative mortality results: randomised controlled trial. Lancet 364: 843–848, 2004 15351191 10.1016/S0140-6736(04)16979-1

[44] Guo L, Akahori H, Harari E, Smith SL, Polavarapu R, Karmali V, Otsuka F, Gannon RL, Braumann RE, Dickinson MH, Gupta A, Jenkins AL, Lipinski MJ, Kim J, Chhour P, de Vries PS, Jinnouchi H, Kutys R, Mori H, Kutyna MD, Torii S, Sakamoto A, Choi CU, Cheng Q, Grove ML, Sawan MA, Zhang Y, Cao Y, Kolodgie FD, Cormode DP, Arking DE, Boerwinkle E, Morrison AC, Erdmann J, Sotoodehnia N, Virmani R, and Finn AV CD163^+^ macrophages promote angiogenesis and vascular permeability accompanied by inflammation in atherosclerosis. J Clin Invest 128: 1106–1124, 2018 29457790 10.1172/JCI93025 PMC5824873

[45] Hance KA, Tataria M, Ziporin SJ, Lee JK, and Thompson RW Monocyte chemotactic activity in human abdominal aortic aneurysms: role of elastin degradation peptides and the 67-kD cell surface elastin receptor. J Vasc Surg 35: 254–261, 2002 11854722 10.1067/mva.2002.120382

[46] Hellenthal FA, Buurman WA, Wodzig WK, and Schurink GW Biomarkers of abdominal aortic aneurysm progression. Part 2: inflammation. Nat Rev Cardiol 6: 543–552, 2009 19546866 10.1038/nrcardio.2009.102

[47] Hinterseher I, Erdman R, Donoso LA, Vrabec TR, Schworer CM, Lillvis JH, Boddy AM, Derr K, Golden A, Bowen WD, Gatalica Z, Tapinos N, Elmore JR, Franklin DP, Gray JL, Garvin RP, Gerhard GS, Carey DJ, Tromp G, and Kuivaniemi H Role of complement cascade in abdominal aortic aneurysms. Arterioscler Thromb Vasc Biol 31: 1653–1660, 2011 21493888 10.1161/ATVBAHA.111.227652 PMC3712630

[48] Houard X, Ollivier V, Louedec L, Michel JB, and Back M Differential inflammatory activity across human abdominal aortic aneurysms reveals neutrophil-derived leukotriene B4 as a major chemotactic factor released from the intraluminal thrombus. FASEB J 23: 1376–1383, 2009 19136615 10.1096/fj.08-116202

[49] Houtkamp MA, de Boer OJ, van der Loos CM, van der Wal AC, and Becker AE Adventitial infiltrates associated with advanced atherosclerotic plaques: structural organization suggests generation of local humoral immune responses. J Pathol 193: 263–269, 2001 11180175 10.1002/1096-9896(2000)9999:9999<::AID-PATH774>3.0.CO;2-N

[50] IMPROVE Trial Investigators, Powell JT, Hinchliffe RJ, Thompson MM, Sweeting MJ, Ashleigh R, Bell R, Gomes M, Greenhalgh RM, Grieve RJ, Heatley F, Thompson SG, and Ulug P Observations from the IMPROVE trial concerning the clinical care of patients with ruptured abdominal aortic aneurysm. Br J Surg 101: 216–224; discussion 224, 2014 10.1002/bjs.9410 PMC4164272 24469620

[51] Jiang L, Qiao Y, Wang Z, Ma X, Wang H, and Li J Inhibition of microRNA-103 attenuates inflammation and endoplasmic reticulum stress in atherosclerosis through disrupting the PTEN-mediated MAPK signaling. J Cell Physiol 235: 380–393, 2020 31232476 10.1002/jcp.28979

[52] Johnston WF, Salmon M, Pope NH, Meher A, Su G, Stone ML, Lu G, Owens GK, Upchurch GR Jr., and Ailawadi G Inhibition of interleukin-1beta decreases aneurysm formation and progression in a novel model of thoracic aortic aneurysms. Circulation 130: S51–S59, 2014 25200056 10.1161/CIRCULATIONAHA.113.006800 PMC5097450

[53] Juvonen J, Surcel HM, Satta J, Teppo AM, Bloigu A, Syrjala H, Airaksinen J, Leinonen M, Saikku P, and Juvonen T Elevated circulating levels of inflammatory cytokines in patients with abdominal aortic aneurysm. Arterioscler Thromb Vasc Biol 17: 2843–2847, 1997 9409264 10.1161/01.atv.17.11.2843

[54] Kaluza J, Stackelberg O, Harris HR, Bjorck M, and Wolk A Anti-inflammatory diet and risk of abdominal aortic aneurysm in two Swedish cohorts. Heart 105: 1876–1883, 2019 31296589 10.1136/heartjnl-2019-315031

[55] Kent KC, Zwolak RM, Egorova NN, Riles TS, Manganaro A, Moskowitz AJ, Gelijns AC, and Greco G Analysis of risk factors for abdominal aortic aneurysm in a cohort of more than 3 million individuals. J Vasc Surg 52: 539–548, 2010 20630687 10.1016/j.jvs.2010.05.090

[56] Kim CW, Kumar S, Son DJ, Jang IH, Griendling KK, and Jo H Prevention of abdominal aortic aneurysm by anti-microRNA-712 or anti-microRNA-205 in angiotensin II-infused mice. Arterioscler Thromb Vasc Biol 34: 1412–1421, 2014 24812324 10.1161/ATVBAHA.113.303134 PMC4111131

[57] Koch AE, Haines GK, Rizzo RJ, Radosevich JA, Pope RM, Robinson PG, and Pearce WH Human abdominal aortic aneurysms. Immunophenotypic analysis suggesting an immune-mediated response. Am J Pathol 137: 1199–1213, 1990 1700620 PMC1877681

[58] Kokje VB, Hamming JF, and Lindeman JH Editor's choice—pharmaceutical management of small abdominal aortic aneurysms: a systematic review of the clinical evidence. Eur J Vasc Endovasc Surg 50: 702–713, 2015 26482507 10.1016/j.ejvs.2015.08.010

[59] Kratofil RM, Kubes P, and Deniset JF Monocyte conversion during inflammation and injury. Arterioscler Thromb Vasc Biol 37: 35–42, 2017 27765768 10.1161/ATVBAHA.116.308198

[60] Kumar S, Boon RA, Maegdefessel L, Dimmeler S, and Jo H Role of noncoding RNAs in the pathogenesis of abdominal aortic aneurysm. Circ Res 124: 619–630, 2019 30763215 10.1161/CIRCRESAHA.118.312438 PMC6440479

[61] Lamblin N, Ratajczak P, Hot D, Dubois E, Chwastyniak M, Beseme O, Drobecq H, Lemoine Y, Koussa M, Amouyel P, and Pinet F Profile of macrophages in human abdominal aortic aneurysms: a transcriptomic, proteomic, and antibody protein array study. J Proteome Res 9: 3720–3729, 2010 20513153 10.1021/pr100250s

[62] Lanone S, Zheng T, Zhu Z, Liu W, Lee CG, Ma B, Chen Q, Homer RJ, Wang J, Rabach LA, Rabach ME, Shipley JM, Shapiro SD, Senior RM, and Elias JA Overlapping and enzyme-specific contributions of matrix metalloproteinases-9 and -12 in IL-13-induced inflammation and remodeling. J Clin Invest 110: 463–474, 2002 12189240 10.1172/JCI14136 PMC150413

[63] LeBien TW and Tedder TF B lymphocytes: how they develop and function. Blood 112: 1570–1580, 2008 18725575 10.1182/blood-2008-02-078071 PMC2518873

[64] Lederle FA. The rise and fall of abdominal aortic aneurysm. Circulation 124: 1097–1099, 2011 21900095 10.1161/CIRCULATIONAHA.111.052365

[65] Lederle FA, Freischlag JA, Kyriakides TC, Padberg FT Jr., Matsumura JS, Kohler TR, Lin PH, Jean-Claude JM, Cikrit DF, Swanson KM, and Peduzzi PN; Open Versus Endovascular Repair (OVER) Veterans Affairs Cooperative Study Group. Outcomes following endovascular vs open repair of abdominal aortic aneurysm: a randomized trial. JAMA 302: 1535–1542, 2009 19826022 10.1001/jama.2009.1426

[66] Lederle FA, Johnson GR, Wilson SE, Ballard DJ, Jordan WD Jr., Blebea J, Littooy FN, Freischlag JA, Bandyk D, Rapp JH, and Salam AA; Veterans Affairs Cooperative Study #417 Investigators. Rupture rate of large abdominal aortic aneurysms in patients refusing or unfit for elective repair. JAMA 287: 2968–2972, 2002 12052126 10.1001/jama.287.22.2968

[67] Lee Y, Ahn C, Han J, Choi H, Kim J, Yim J, Lee J, Provost P, Radmark O, Kim S, and Kim VN The nuclear RNase III Drosha initiates microRNA processing. Nature 425: 415–419, 2003 14508493 10.1038/nature01957

[68] Leeper NJ and Maegdefessel L Non-coding RNAs: key regulators of smooth muscle cell fate in vascular disease. Cardiovasc Res 114: 611–621, 2018 29300828 10.1093/cvr/cvx249 PMC5852528

[69] Leskinen M, Wang Y, Leszczynski D, Lindstedt KA, and Kovanen PT Mast cell chymase induces apoptosis of vascular smooth muscle cells. Arterioscler Thromb Vasc Biol 21: 516–522, 2001 11304466 10.1161/01.atv.21.4.516

[70] Leung A, Trac C, Jin W, Lanting L, Akbany A, Saetrom P, Schones DE, and Natarajan R Novel long noncoding RNAs are regulated by angiotensin II in vascular smooth muscle cells. Circ Res 113: 266–278, 2013 23697773 10.1161/CIRCRESAHA.112.300849 PMC3763837

[71] Li DY, Busch A, Jin H, Chernogubova E, Pelisek J, Karlsson J, Sennblad B, Liu S, Lao S, Hofmann P, Backlund A, Eken SM, Roy J, Eriksson P, Dacken B, Ramanujam D, Dueck A, Engelhardt S, Boon RA, Eckstein HH, Spin JM, Tsao PS, and Maegdefessel L H19 induces abdominal aortic aneurysm development and progression. Circulation 138: 1551–1568, 2018 29669788 10.1161/CIRCULATIONAHA.117.032184 PMC6193867

[72] Li P, Zhu N, Yi B, Wang N, Chen M, You X, Zhao X, Solomides CC, Qin Y, and Sun J MicroRNA-663 regulates human vascular smooth muscle cell phenotypic switch and vascular neointimal formation. Circ Res 113: 1117–1127, 2013 24014830 10.1161/CIRCRESAHA.113.301306 PMC4537615

[73] Li Y, Liu Y, Liu S, Wu F, Li S, Yang F, Gu Y, Xu Z, and Wang G Differential expression profile of long non-coding RNAs in human thoracic aortic aneurysm. J Cell Biochem 119: 7991–7997, 2018 29323743 10.1002/jcb.26670

[74] Li Y and Maegdefessel L Non-coding RNA contribution to thoracic and abdominal aortic aneurysm disease development and progression. Front Physiol 8: 429, 2017 28670289 10.3389/fphys.2017.00429 PMC5472729

[75] Li Z, Chao TC, Chang KY, Lin N, Patil VS, Shimizu C, Head SR, Burns JC, and Rana TM The long noncoding RNA THRIL regulates TNFalpha expression through its interaction with hnRNPL. Proc Natl Acad Sci U S A 111: 1002–1007, 2014 24371310 10.1073/pnas.1313768111 PMC3903238

[76] Lin X, He Y, Hou X, Zhang Z, Wang R, and Wu Q Endothelial cells can regulate smooth muscle cells in contractile phenotype through the miR-206/ARF6&NCX1/exosome axis. PLoS One 11: e0152959, 2016 27031991 10.1371/journal.pone.0152959 PMC4816502

[77] Lindholt JS, Stovring J, Ostergaard L, Urbonavicius S, Henneberg EW, Honore B, and Vorum H Serum antibodies against Chlamydia pneumoniae outer membrane protein cross-react with the heavy chain of immunoglobulin in the wall of abdominal aortic aneurysms. Circulation 109: 2097–2102, 2004 15117850 10.1161/01.CIR.0000127772.58427.7E

[78] Lu TX, Munitz A, and Rothenberg ME MicroRNA-21 is up-regulated in allergic airway inflammation and regulates IL-12p35 expression. J Immunol 182: 4994–5002, 2009 19342679 10.4049/jimmunol.0803560 PMC4280862

[79] Madhur MS, Funt SA, Li L, Vinh A, Chen W, Lob HE, Iwakura Y, Blinder Y, Rahman A, Quyyumi AA, and Harrison DG Role of interleukin 17 in inflammation, atherosclerosis, and vascular function in apolipoprotein e-deficient mice. Arterioscler Thromb Vasc Biol 31: 1565–1572, 2011 21474820 10.1161/ATVBAHA.111.227629 PMC3117048

[80] Maegdefessel L, Azuma J, Toh R, Deng A, Merk DR, Raiesdana A, Leeper NJ, Raaz U, Schoelmerich AM, McConnell MV, Dalman RL, Spin JM, and Tsao PS MicroRNA-21 blocks abdominal aortic aneurysm development and nicotine-augmented expansion. Sci Transl Med 4: 122ra22, 2012 10.1126/scitranslmed.3003441 PMC5753594 22357537

[81] Maegdefessel L, Azuma J, Toh R, Merk DR, Deng A, Chin JT, Raaz U, Schoelmerich AM, Raiesdana A, Leeper NJ, McConnell MV, Dalman RL, Spin JM, and Tsao PS Inhibition of microRNA-29b reduces murine abdominal aortic aneurysm development. J Clin Invest 122: 497–506, 2012 22269326 10.1172/JCI61598 PMC3266800

[82] Maegdefessel L, Spin JM, Raaz U, Eken SM, Toh R, Azuma J, Adam M, Nakagami F, Heymann HM, Chernogubova E, Jin H, Roy J, Hultgren R, Caidahl K, Schrepfer S, Hamsten A, Eriksson P, McConnell MV, Dalman RL, and Tsao PS miR-24 limits aortic vascular inflammation and murine abdominal aneurysm development. Nat Commun 5: 5214, 2014 25358394 10.1038/ncomms6214 PMC4217126

[83] Mathy NW and Chen XM Long non-coding RNAs (lncRNAs) and their transcriptional control of inflammatory responses. J Biol Chem 292: 12375–12382, 2017 28615453 10.1074/jbc.R116.760884 PMC5535013

[84] Meher AK, Johnston WF, Lu G, Pope NH, Bhamidipati CM, Harmon DB, Su G, Zhao Y, McNamara CA, Upchurch GR Jr., and Ailawadi G B2 cells suppress experimental abdominal aortic aneurysms. Am J Pathol 184: 3130–3141, 2014 25194661 10.1016/j.ajpath.2014.07.006 PMC4215033

[85] Meher AK, Spinosa M, Davis JP, Pope N, Laubach VE, Su G, Serbulea V, Leitinger N, Ailawadi G, and Upchurch GR Jr Novel role of IL (interleukin)-1beta in neutrophil extracellular trap formation and abdominal aortic aneurysms. Arterioscler Thromb Vasc Biol 38: 843–853, 2018 29472233 10.1161/ATVBAHA.117.309897 PMC5864548

[86] Mellak S, Ait-Oufella H, Esposito B, Loyer X, Poirier M, Tedder TF, Tedgui A, Mallat Z, and Potteaux S Angiotensin II mobilizes spleen monocytes to promote the development of abdominal aortic aneurysm in ApoE^−/−^ mice. Arterioscler Thromb Vasc Biol 35: 378–388, 2015 25524776 10.1161/ATVBAHA.114.304389

[87] Merk DR, Chin JT, Dake BA, Maegdefessel L, Miller MO, Kimura N, Tsao PS, Iosef C, Berry GJ, Mohr FW, Spin JM, Alvira CM, Robbins RC, and Fischbein MP miR-29b participates in early aneurysm development in Marfan syndrome. Circ Res 110: 312–324, 2012 22116819 10.1161/CIRCRESAHA.111.253740

[88] Middleton RK, Lloyd GM, Bown MJ, Cooper NJ, London NJ, and Sayers RD The pro-inflammatory and chemotactic cytokine microenvironment of the abdominal aortic aneurysm wall: a protein array study. J Vasc Surg 45: 574–580, 2007 17321344 10.1016/j.jvs.2006.11.020

[89] Moehle CW, Bhamidipati CM, Alexander MR, Mehta GS, Irvine JN, Salmon M, Upchurch GR Jr., Kron IL, Owens GK, and Ailawadi G. Bone marrow-derived MCP1 required for experimental aortic aneurysm formation and smooth muscle phenotypic modulation. J Thorac Cardiovasc Surg 142: 1567–1574, 2011 21996300 10.1016/j.jtcvs.2011.07.053 PMC3627218

[90] Moran CS, Jose RJ, Moxon JV, Roomberg A, Norman PE, Rush C, Korner H, and Golledge J Everolimus limits aortic aneurysm in the apolipoprotein E-deficient mouse by downregulating C-C chemokine receptor 2 positive monocytes. Arterioscler Thromb Vasc Biol 33: 814–821, 2013 23393391 10.1161/ATVBAHA.112.301006

[91] Murray PJ, Allen JE, Biswas SK, Fisher EA, Gilroy DW, Goerdt S, Gordon S, Hamilton JA, Ivashkiv LB, Lawrence T, Locati M, Mantovani A, Martinez FO, Mege JL, Mosser DM, Natoli G, Saeij JP, Schultze JL, Shirey KA, Sica A, Suttles J, Udalova I, van Ginderachter JA, Vogel SN, and Wynn TA Macrophage activation and polarization: nomenclature and experimental guidelines. Immunity 41: 14–20, 2014 25035950 10.1016/j.immuni.2014.06.008 PMC4123412

[92] Nakao T, Horie T, Baba O, Nishiga M, Nishino T, Izuhara M, Kuwabara Y, Nishi H, Usami S, Nakazeki F, Ide Y, Koyama S, Kimura M, Sowa N, Ohno S, Aoki H, Hasegawa K, Sakamoto K, Minatoya K, Kimura T, and Ono K Genetic ablation of microRNA-33 attenuates inflammation and abdominal aortic aneurysm formation via several anti-inflammatory pathways. Arterioscler Thromb Vasc Biol 37: 2161–2170, 2017 28882868 10.1161/ATVBAHA.117.309768

[93] Nalewajska M, Gurazda K, Styczynska-Kowalska E, Marchelek-Mysliwiec M, Pawlik A, and Dziedziejko V. The role of microRNAs in selected forms of glomerulonephritis. Int J Mol Sci 20, 2019 10.3390/ijms20205050 PMC6834307 31614644

[94] Naqvi AR, Fordham JB, and Nares S miR-24, miR-30b, and miR-142-3p regulate phagocytosis in myeloid inflammatory cells. J Immunol 194: 1916–1927, 2015 25601927 10.4049/jimmunol.1401893 PMC4323870

[95] Nazari-Jahantigh M, Wei Y, Noels H, Akhtar S, Zhou Z, Koenen RR, Heyll K, Gremse F, Kiessling F, Grommes J, Weber C, and Schober A MicroRNA-155 promotes atherosclerosis by repressing Bcl6 in macrophages. J Clin Invest 122: 4190–4202, 2012 23041630 10.1172/JCI61716 PMC3484435

[96] Pagano MB, Zhou HF, Ennis TL, Wu X, Lambris JD, Atkinson JP, Thompson RW, Hourcade DE, and Pham CT Complement-dependent neutrophil recruitment is critical for the development of elastase-induced abdominal aortic aneurysm. Circulation 119: 1805–1813, 2009 19307471 10.1161/CIRCULATIONAHA.108.832972 PMC2758616

[97] Pan JH, Lindholt JS, Sukhova GK, Baugh JA, Henneberg EW, Bucala R, Donnelly SC, Libby P, Metz C, and Shi GP Macrophage migration inhibitory factor is associated with aneurysmal expansion. J Vasc Surg 37: 628–635, 2003 12618703 10.1067/mva.2003.74

[98] Patel R, Sweeting MJ, Powell JT, and Greenhalgh RM; EVAR Trial Investigators Endovascular versus open repair of abdominal aortic aneurysm in 15-years' follow-up of the UK endovascular aneurysm repair trial 1 (EVAR trial 1): a randomised controlled trial. Lancet 388: 2366–2374, 2016 27743617 10.1016/S0140-6736(16)31135-7

[99] Pearson MJ, Philp AM, Heward JA, Roux BT, Walsh DA, Davis ET, Lindsay MA, and Jones SW Long intergenic noncoding RNAs mediate the human chondrocyte inflammatory response and are differentially expressed in osteoarthritis cartilage. Arthritis Rheumatol 68: 845–856, 2016 27023358 10.1002/art.39520 PMC4950001

[100] Perdiguero EG and Geissmann F The development and maintenance of resident macrophages. Nat Immunol 17: 2–8, 2016 26681456 10.1038/ni.3341 PMC4950995

[101] Piechota-Polanczyk A, Jozkowicz A, Nowak W, Eilenberg W, Neumayer C, Malinski T, Huk I, and Brostjan C The abdominal aortic aneurysm and intraluminal thrombus: current concepts of development and treatment. Front Cardiovasc Med 2: 19, 2015 26664891 10.3389/fcvm.2015.00019 PMC4671358

[102] Powell JT, Brown LC, Forbes JF, Fowkes FG, Greenhalgh RM, Ruckley CV, and Thompson SG Final 12-year follow-up of surgery versus surveillance in the UK Small Aneurysm Trial. Br J Surg 94: 702–708, 2007 17514693 10.1002/bjs.5778

[103] Prinssen M, Verhoeven EL, Buth J, Cuypers PW, van Sambeek MR, Balm R, Buskens E, Grobbee DE, and Blankensteijn JD; Dutch Randomized Endovascular Aneurysm Management (DREAM) Trial Group A randomized trial comparing conventional and endovascular repair of abdominal aortic aneurysms. N Engl J Med 351: 1607–1618, 2004 15483279 10.1056/NEJMoa042002

[104] Pyo R, Lee JK, Shipley JM, Curci JA, Mao D, Ziporin SJ, Ennis TL, Shapiro SD, Senior RM, and Thompson RW Targeted gene disruption of matrix metalloproteinase-9 (gelatinase B) suppresses development of experimental abdominal aortic aneurysms. J Clin Invest 105: 1641–1649, 2000 10841523 10.1172/JCI8931 PMC300851

[105] Qin Z, Bagley J, Sukhova G, Baur WE, Park HJ, Beasley D, Libby P, Zhang Y, and Galper JB Angiotensin II-induced TLR4 mediated abdominal aortic aneurysm in apolipoprotein E knockout mice is dependent on STAT3. J Mol Cell Cardiol 87: 160–170, 2015 26299839 10.1016/j.yjmcc.2015.08.014

[106] Raffort J, Lareyre F, Clement M, Hassen-Khodja R, Chinetti G, and Mallat Z Monocytes and macrophages in abdominal aortic aneurysm. Nat Rev Cardiol 14: 457–471, 2017 28406184 10.1038/nrcardio.2017.52

[107] Ramos-Mozo P, Madrigal-Matute J, Martinez-Pinna R, Blanco-Colio LM, Lopez JA, Camafeita E, Meilhac O, Michel JB, Aparicio C, Vega de Ceniga M, Egido J, and Martin-Ventura JL Proteomic analysis of polymorphonuclear neutrophils identifies catalase as a novel biomarker of abdominal aortic aneurysm: potential implication of oxidative stress in abdominal aortic aneurysm progression. Arterioscler Thromb Vasc Biol 31: 3011–3019, 2011 21940941 10.1161/ATVBAHA.111.237537

[108] Rao J, Brown BN, Weinbaum JS, Ofstun EL, Makaroun MS, Humphrey JD, and Vorp DA Distinct macrophage phenotype and collagen organization within the intraluminal thrombus of abdominal aortic aneurysm. J Vasc Surg 62: 585–593, 2015 26206580 10.1016/j.jvs.2014.11.086 PMC4550501

[109] Rateri DL, Howatt DA, Moorleghen JJ, Charnigo R, Cassis LA, and Daugherty A Prolonged infusion of angiotensin II in apoE(−/−) mice promotes macrophage recruitment with continued expansion of abdominal aortic aneurysm. Am J Pathol 179: 1542–1548, 2011 21763672 10.1016/j.ajpath.2011.05.049 PMC3157213

[110] Rayner KJ, Suarez Y, Davalos A, Parathath S, Fitzgerald ML, Tamehiro N, Fisher EA, Moore KJ, and Fernandez-Hernando C MiR-33 contributes to the regulation of cholesterol homeostasis. Science 328: 1570–1573, 2010 20466885 10.1126/science.1189862 PMC3114628

[111] Rong JX, Shapiro M, Trogan E, and Fisher EA Transdifferentiation of mouse aortic smooth muscle cells to a macrophage-like state after cholesterol loading. Proc Natl Acad Sci U S A 100: 13531–13536, 2003 14581613 10.1073/pnas.1735526100 PMC263848

[112] Rubio-Navarro A, Amaro Villalobos JM, Lindholt JS, Buendia I, Egido J, Blanco-Colio LM, Samaniego R, Meilhac O, Michel JB, Martin-Ventura JL, and Moreno JA Hemoglobin induces monocyte recruitment and CD163-macrophage polarization in abdominal aortic aneurysm. Int J Cardiol 201: 66–78, 2015 26296046 10.1016/j.ijcard.2015.08.053

[113] Rupaimoole R and Slack FJ MicroRNA therapeutics: towards a new era for the management of cancer and other diseases. Nat Rev Drug Discov 16: 203–222, 2017 28209991 10.1038/nrd.2016.246

[114] Salata K, Syed M, Hussain MA, de Mestral C, Greco E, Mamdani M, Tu JV, Forbes TL, Bhatt DL, Verma S, and Al-Omran M Statins reduce abdominal aortic aneurysm growth, rupture, and perioperative mortality: a systematic review and meta-analysis. J Am Heart Assoc 7: e008657, 2018 30371297 10.1161/JAHA.118.008657 PMC6404894

[115] Samadzadeh KM, Chun KC, Nguyen AT, Baker PM, Bains S, and Lee ES Monocyte activity is linked with abdominal aortic aneurysm diameter. J Surg Res 190: 328–334, 2014 24726061 10.1016/j.jss.2014.03.019

[116] Schonbeck U, Sukhova GK, Gerdes N, and Libby P T(H)2 predominant immune responses prevail in human abdominal aortic aneurysm. Am J Pathol 161: 499–506, 2002 12163375 10.1016/S0002-9440(10)64206-X PMC1850720

[117] Shafiani S, Tucker-Heard G, Kariyone A, Takatsu K, and Urdahl KB Pathogen-specific regulatory T cells delay the arrival of effector T cells in the lung during early tuberculosis. J Exp Med 207: 1409–1420, 2010 20547826 10.1084/jem.20091885 PMC2901066

[118] Shankman LS, Gomez D, Cherepanova OA, Salmon M, Alencar GF, Haskins RM, Swiatlowska P, Newman AA, Greene ES, Straub AC, Isakson B, Randolph GJ, and Owens GK KLF4-dependent phenotypic modulation of smooth muscle cells has a key role in atherosclerotic plaque pathogenesis. Nat Med 21: 628–637, 2015 25985364 10.1038/nm.3866 PMC4552085

[119] Sharma AK, Lu G, Jester A, Johnston WF, Zhao Y, Hajzus VA, Saadatzadeh MR, Su G, Bhamidipati CM, Mehta GS, Kron IL, Laubach VE, Murphy MP, Ailawadi G, and Upchurch GR Jr Experimental abdominal aortic aneurysm formation is mediated by IL-17 and attenuated by mesenchymal stem cell treatment. Circulation 126: S38–S45, 2012 22965992 10.1161/CIRCULATIONAHA.111.083451 PMC3448933

[120] Shimizu K, Shichiri M, Libby P, Lee RT, and Mitchell RN Th2-predominant inflammation and blockade of IFN-gamma signaling induce aneurysms in allografted aortas. J Clin Invest 114: 300–308, 2004 15254597 10.1172/JCI19855 PMC449742

[121] Sillesen H, Eldrup N, Hultgren R, Lindeman J, Bredahl K, Thompson M, Wanhainen A, Wingren U, and Swedenborg J; AORTA Trial Investigators Randomized clinical trial of mast cell inhibition in patients with a medium-sized abdominal aortic aneurysm. Br J Surg 102: 894–901, 2015 25963302 10.1002/bjs.9824

[122] Song J, Yang S, Yin R, Xiao Q, Ma A, and Pan X MicroRNA-181a regulates the activation of the NLRP3 inflammatory pathway by targeting MEK1 in THP-1 macrophages stimulated by ox-LDL. J Cell Biochem 120: 13640–13650, 2019 30938884 10.1002/jcb.28637

[123] Sonkoly E and Pivarcsi A microRNAs in inflammation. Int Rev Immunol 28: 535–561, 2009 19954362 10.3109/08830180903208303

[124] Sonkoly E, Wei T, Janson PC, Saaf A, Lundeberg L, Tengvall-Linder M, Norstedt G, Alenius H, Homey B, Scheynius A, Stahle M, and Pivarcsi A MicroRNAs: novel regulators involved in the pathogenesis of psoriasis? PLoS One 2: e610, 2007 17622355 10.1371/journal.pone.0000610 PMC1905940

[125] Spurlock CF, 3rd, Tossberg JT, Matlock BK, Olsen NJ, and Aune TM Methotrexate inhibits NF-kappaB activity via long intergenic (noncoding) RNA-p21 induction. Arthritis Rheumatol 66: 2947–2957, 2014 25077978 10.1002/art.38805 PMC4211976

[126] Stanczyk J, Pedrioli DM, Brentano F, Sanchez-Pernaute O, Kolling C, Gay RE, Detmar M, Gay S, and Kyburz D Altered expression of microRNA in synovial fibroblasts and synovial tissue in rheumatoid arthritis. Arthritis Rheum 58: 1001–1009, 2008 18383392 10.1002/art.23386

[127] Su Y, Yuan J, Zhang F, Lei Q, Zhang T, Li K, Guo J, Hong Y, Bu G, Lv X, Liang S, Ou J, Zhou J, Luo B, and Shang J MicroRNA-181a-5p and microRNA-181a-3p cooperatively restrict vascular inflammation and atherosclerosis. Cell Death Dis 10: 365, 2019 31064980 10.1038/s41419-019-1599-9 PMC6504957

[128] Sun SG, Zheng B, Han M, Fang XM, Li HX, Miao SB, Su M, Han Y, Shi HJ, and Wen JK miR-146a and Kruppel-like factor 4 form a feedback loop to participate in vascular smooth muscle cell proliferation. EMBO Rep 12: 56–62, 2011 21109779 10.1038/embor.2010.172 PMC3024123

[129] Svensjo S, Bjorck M, Gurtelschmid M, Djavani Gidlund K, Hellberg A, and Wanhainen A Low prevalence of abdominal aortic aneurysm among 65-year-old Swedish men indicates a change in the epidemiology of the disease. Circulation 124: 1118–1123, 2011 21844079 10.1161/CIRCULATIONAHA.111.030379

[130] Swirski FK, Libby P, Aikawa E, Alcaide P, Luscinskas FW, Weissleder R, and Pittet MJ Ly-6Chi monocytes dominate hypercholesterolemia-associated monocytosis and give rise to macrophages in atheromata. J Clin Invest 117: 195–205, 2007 17200719 10.1172/JCI29950 PMC1716211

[131] Tabas I and Bornfeldt KE Macrophage Phenotype and Function in Different Stages of Atherosclerosis. Circ Res 118: 653–667, 2016 26892964 10.1161/CIRCRESAHA.115.306256 PMC4762068

[132] Tacke F, Alvarez D, Kaplan TJ, Jakubzick C, Spanbroek R, Llodra J, Garin A, Liu J, Mack M, van Rooijen N, Lira SA, Habenicht AJ, and Randolph GJ Monocyte subsets differentially employ CCR2, CCR5, and CX_3_CR1 to accumulate within atherosclerotic plaques. J Clin Invest 117: 185–194, 2007 17200718 10.1172/JCI28549 PMC1716202

[133] Tang Y, Luo X, Cui H, Ni X, Yuan M, Guo Y, Huang X, Zhou H, de Vries N, Tak PP, Chen S, and Shen N MicroRNA-146A contributes to abnormal activation of the type I interferon pathway in human lupus by targeting the key signaling proteins. Arthritis Rheum 60: 1065–1075, 2009 19333922 10.1002/art.24436

[134] Tay C, Liu YH, Hosseini H, Kanellakis P, Cao A, Peter K, Tipping P, Bobik A, Toh BH, and Kyaw T B-cell-specific depletion of tumour necrosis factor alpha inhibits atherosclerosis development and plaque vulnerability to rupture by reducing cell death and inflammation. Cardiovasc Res 111: 385–397, 2016 27492217 10.1093/cvr/cvw186

[135] Tchougounova E, Lundequist A, Fajardo I, Winberg JO, Abrink M, and Pejler G A key role for mast cell chymase in the activation of pro-matrix metalloprotease-9 and pro-matrix metalloprotease-2. J Biol Chem 280: 9291–9296, 2005 15615702 10.1074/jbc.M410396200

[136] Tieu BC, Lee C, Sun H, Lejeune W, Recinos A, 3rd, Ju X, Spratt H, Guo DC, Milewicz D, Tilton RG, and Brasier AR An adventitial IL-6/MCP1 amplification loop accelerates macrophage-mediated vascular inflammation leading to aortic dissection in mice. J Clin Invest 119: 3637–3651, 2009 19920349 10.1172/JCI38308 PMC2786788

[137] Tsuruda T, Kato J, Hatakeyama K, Kojima K, Yano M, Yano Y, Nakamura K, Nakamura-Uchiyama F, Matsushima Y, Imamura T, Onitsuka T, Asada Y, Nawa Y, Eto T, and Kitamura K Adventitial mast cells contribute to pathogenesis in the progression of abdominal aortic aneurysm. Circ Res 102: 1368–1377, 2008 18451339 10.1161/CIRCRESAHA.108.173682

[138] van Schaik TG, Yeung KK, Verhagen HJ, de Bruin JL, van Sambeek M, Balm R, Zeebregts CJ, van Herwaarden JA, and Blankensteijn JD; DREAM Trial Participants Long-term survival and secondary procedures after open or endovascular repair of abdominal aortic aneurysms. J Vasc Surg 66: 1379–1389, 2017 29061270 10.1016/j.jvs.2017.05.122

[139] Vengrenyuk Y, Nishi H, Long X, Ouimet M, Savji N, Martinez FO, Cassella CP, Moore KJ, Ramsey SA, Miano JM, and Fisher EA Cholesterol loading reprograms the microRNA-143/145-myocardin axis to convert aortic smooth muscle cells to a dysfunctional macrophage-like phenotype. Arterioscler Thromb Vasc Biol 35: 535–546, 2015 25573853 10.1161/ATVBAHA.114.304029 PMC4344402

[140] Wamhoff BR, Hoofnagle MH, Burns A, Sinha S, McDonald OG, and Owens GK A G/C element mediates repression of the SM22alpha promoter within phenotypically modulated smooth muscle cells in experimental atherosclerosis. Circ Res 95: 981–988, 2004 15486317 10.1161/01.RES.0000147961.09840.fb

[141] Wanhainen A, Mani K, Vorkapic E, De Basso R, Bjorck M, Lanne T, and Wagsater D Screening of circulating microRNA biomarkers for prevalence of abdominal aortic aneurysm and aneurysm growth. Atherosclerosis 256: 82–88, 2017 27993388 10.1016/j.atherosclerosis.2016.11.007

[142] Wanhainen A, Verzini F, Van Herzeele I, Allaire E, Bown M, Cohnert T, Dick F, van Herwaarden J, Karkos C, Koelemay M, Kolbel T, Loftus I, Mani K, Melissano G, Powell J, Szeberin Z, Esvs Guidelines Committee, de Borst GJ, Chakfe N, Debus S, Hinchliffe R, Kakkos S, Koncar I, Kolh P, Lindholt JS, de Vega M, Vermassen F, Document R, Bjorck M, Cheng S, Dalman R, Davidovic L, Donas K, Earnshaw J, Eckstein HH, Golledge J, Haulon S, Mastracci T, Naylor R, Ricco JB, and Verhagen H Editor's choice—European Society for Vascular Surgery (ESVS) 2019 clinical practice guidelines on the management of abdominal aorto-iliac artery aneurysms. Eur J Vasc Endovasc Surg 57: 8–93, 2019 30528142 10.1016/j.ejvs.2018.09.020

[143] Wei Y, Nazari-Jahantigh M, Chan L, Zhu M, Heyll K, Corbalan-Campos J, Hartmann P, Thiemann A, Weber C, and Schober A The microRNA-342-5p fosters inflammatory macrophage activation through an Akt1- and microRNA-155-dependent pathway during atherosclerosis. Circulation 127: 1609–1619, 2013 23513069 10.1161/CIRCULATIONAHA.112.000736

[144] Wei Y, Zhu M, and Schober A. Macrophage microRNAs as therapeutic targets for atherosclerosis, metabolic syndrome, and cancer. Int J Mol Sci 19, 2018 10.3390/ijms19061756 PMC6032097 29899293

[145] Wick G, Romen M, Amberger A, Metzler B, Mayr M, Falkensammer G, and Xu Q Atherosclerosis, autoimmunity, and vascular-associated lymphoid tissue. FASEB J 11: 1199–1207, 1997 9367355 10.1096/fasebj.11.13.9367355

[146] Wong KL, Tai JJ, Wong WC, Han H, Sem X, Yeap WH, Kourilsky P, and Wong SC Gene expression profiling reveals the defining features of the classical, intermediate, and nonclassical human monocyte subsets. Blood 118: e16–e31, 2011 21653326 10.1182/blood-2010-12-326355

[147] Wu F, Zikusoka M, Trindade A, Dassopoulos T, Harris ML, Bayless TM, Brant SR, Chakravarti S, and Kwon JH MicroRNAs are differentially expressed in ulcerative colitis and alter expression of macrophage inflammatory peptide-2 alpha. Gastroenterology 135: 1624.e24–1635.e24, 2008 10.1053/j.gastro.2008.07.068 18835392

[148] Wu G, Chen T, Shahsafaei A, Hu W, Bronson RT, Shi GP, Halperin JA, Aktas H, and Qin X Complement regulator CD59 protects against angiotensin II-induced abdominal aortic aneurysms in mice. Circulation 121: 1338–1346, 2010 20212283 10.1161/CIRCULATIONAHA.108.844589 PMC3057574

[149] Xia S, Ozsvath K, Hirose H, and Tilson MD Partial amino acid sequence of a novel 40-kDa human aortic protein, with vitronectin-like, fibrinogen-like, and calcium binding domains: aortic aneurysm-associated protein-40 (AAAP-40) [human MAGP-3, proposed]. Biochem Biophys Res Commun 219: 36–39, 1996 8619823 10.1006/bbrc.1996.0177

[150] Xiong W, MacTaggart J, Knispel R, Worth J, Persidsky Y, and Baxter BT Blocking TNF-alpha attenuates aneurysm formation in a murine model. J Immunol 183: 2741–2746, 2009 19620291 10.4049/jimmunol.0803164 PMC4028114

[151] Xiong W, Zhao Y, Prall A, Greiner TC, and Baxter BT Key roles of CD4^+^ T cells and IFN-gamma in the development of abdominal aortic aneurysms in a murine model. J Immunol 172: 2607–2612, 2004 14764734 10.4049/jimmunol.172.4.2607

[152] Yin M, Zhang J, Wang Y, Wang S, Bockler D, Duan Z, and Xin S Deficient CD4^+^CD25^+^ T regulatory cell function in patients with abdominal aortic aneurysms. Arterioscler Thromb Vasc Biol 30: 1825–1831, 2010 20448211 10.1161/ATVBAHA.109.200303

[153] Ying H, Kang Y, Zhang H, Zhao D, Xia J, Lu Z, Wang H, Xu F, and Shi L MiR-127 modulates macrophage polarization and promotes lung inflammation and injury by activating the JNK pathway. J Immunol 194: 1239–1251, 2015 25520401 10.4049/jimmunol.1402088

[154] Zampetaki A, Attia R, Mayr U, Gomes RS, Phinikaridou A, Yin X, Langley SR, Willeit P, Lu R, Fanshawe B, Fava M, Barallobre-Barreiro J, Molenaar C, So PW, Abbas A, Jahangiri M, Waltham M, Botnar R, Smith A, and Mayr M Role of miR-195 in aortic aneurysmal disease. Circ Res 115: 857–866, 2014 25201911 10.1161/CIRCRESAHA.115.304361

[155] Zhang F, Liu G, Wei C, Gao C, and Hao J Linc-MAF-4 regulates Th1/Th2 differentiation and is associated with the pathogenesis of multiple sclerosis by targeting MAF. FASEB J 31: 519–525, 2017 27756768 10.1096/fj.201600838R

[156] Zhang L and Wang Y B lymphocytes in abdominal aortic aneurysms. Atherosclerosis 242: 311–317, 2015 26233918 10.1016/j.atherosclerosis.2015.07.036

[157] Zheng L, Xu CC, Chen WD, Shen WL, Ruan CC, Zhu LM, Zhu DL, and Gao PJ MicroRNA-155 regulates angiotensin II type 1 receptor expression and phenotypic differentiation in vascular adventitial fibroblasts. Biochem Biophys Res Commun 400: 483–488, 2010 20735984 10.1016/j.bbrc.2010.08.067

[158] Zhou HF, Yan H, Stover CM, Fernandez TM, Rodriguez de Cordoba S, Song WC, Wu X, Thompson RW, Schwaeble WJ, Atkinson JP, Hourcade DE, and Pham CT Antibody directs properdin-dependent activation of the complement alternative pathway in a mouse model of abdominal aortic aneurysm. Proc Natl Acad Sci U S A 109: E415–E422, 2012 22308431 10.1073/pnas.1119000109 PMC3289386

[159] Zhu J and Paul WE CD4 T cells: fates, functions, and faults. Blood 112: 1557–1569, 2008 18725574 10.1182/blood-2008-05-078154 PMC2518872

[160] Ziegler-Heitbrock L. Blood monocytes and their subsets: established features and open questions. Front Immunol 6: 423, 2015 26347746 10.3389/fimmu.2015.00423 PMC4538304

